# Genomic Analyses Reveal Adaptation to Hot Arid and Harsh Environments in Native Chickens of China

**DOI:** 10.3389/fgene.2020.582355

**Published:** 2020-12-21

**Authors:** Jingjing Gu, Qiqi Liang, Can Liu, Sheng Li

**Affiliations:** ^1^College of Animal Science and Technology, Hunan Agricultural University, Changsha, China; ^2^Hunan Provincial Key Laboratory for Genetic Improvement of Domestic Animal, Changsha, China; ^3^Hunan Engineering Research Center of Poultry Production Safety, Changsha, China; ^4^Novogene Bioinformatics Institute, Beijing, China; ^5^Maxun Biotechnology Institute, Changsha, China

**Keywords:** positive selection, native chicken, environmental adaption, whole genome resequencing, candidate genes

## Abstract

The acute thermal response has been extensively studied in commercial chickens because of the adverse effects of heat stress on poultry production worldwide. Here, we performed whole-genome resequencing of autochthonous Niya chicken breed native to the Taklimakan Desert region as well as of 11 native chicken breeds that are widely distributed and reared under native humid and temperate areas. We used combined statistical analysis to search for putative genes that might be related to the adaptation of hot arid and harsh environment in Niya chickens. We obtained a list of intersected candidate genes with log2 θπ ratio, FST and XP-CLR (including 123 regions of 21 chromosomes with the average length of 54.4 kb) involved in different molecular processes and pathways implied complex genetic mechanisms of adaptation of native chickens to hot arid and harsh environments. We identified several selective regions containing genes that were associated with the circulatory system and blood vessel development (BVES, SMYD1, IL18, PDGFRA, NRP1, and CORIN), related to central nervous system development (SIM2 and NALCN), related to apoptosis (CLPTM1L, APP, CRADD, and PARK2) responded to stimuli (AHR, ESRRG FAS, and UBE4B) and involved in fatty acid metabolism (FABP1). Our findings provided the genomic evidence of the complex genetic mechanisms of adaptation to hot arid and harsh environments in chickens. These results may improve our understanding of thermal, drought, and harsh environment acclimation in chickens and may serve as a valuable resource for developing new biotechnological tools to breed stress-tolerant chicken lines and or breeds in the future.

## Introduction

Domestic chickens (*Gallus gallus* domesticus) were most likely domesticated from wild Red Jungle Fowls (Miao et al., 2013; Wang M.-S. et al., 2020) and became the most abundant domestic animals all over the world (Lawler, 2014). After a long period of artificial and natural selection, over hundreds of distinct chicken breeds exist and thrive, and which could be raised in various geographical environments. Furthermore, considering the characteristics of short reproductive and growth periods and large clutch sizes, chickens can be used as ideal models to study genetic adaptations to environments. The chicken genome has been *de novo* assembled (Hillier et al., 2004; Warren et al., 2017) which allowed us to access whole-genome information to detect the genetic signatures of selection left in the genome.

Due to the effects of common global environmental stressors (e.g., heat stress) on poultry production, it is important to improve our understanding of the genetic mechanisms associated with the negative effects of stressful environments and conditions. Although the acute heat response has been extensively studied in commercial chickens because of the adverse effects of heat stress on poultry production worldwide (Nawab et al., 2018) and some studies have been done to exam the environmental adaptability of native chickens (Elbeltagy et al., 2019; Walugembe et al., 2019; Wang Q. et al., 2020), there are short of reports on the genetic mechanisms of domestic chickens adapting to naturally occurring hot arid and harsh environments with multiple stressors (e.g., heat stress, drought, and other adverse stimuli) in China.

The Niya chicken (NY) is a native Chinese chicken breed that originated from the northwest region of the Tarim Basin, the southern margin of the Taklimakan Desert. The living environment of NY belongs to the temperate desert climate zone with high solar radiation, huge diurnal ambient temperature difference, high daytime temperatures, frequent floating dust weather (>220 days/year), extremely low rainfall (30.2 mm/year), and high evaporation (2756.1–2,824 mm/year). While NYs have always been raised like free-range chickens outside by local people, they have encountered more drought and harsher conditions than their counterparts (Table 1) living in humid and temperate areas.

**Table 1:** Comparison of living environment differences among different chicken breeds.

**Individual name*^a^***	**Breeds**	**Geographic locations**	**Annual precipitation (mm)*^b^***	**Average Annual Temperature (^**°**^C)*^b^***	**Frost-free period of the year (days)*^b^***	**The annual sunshine duration (h)*^b^***	**Environment characteristics**
DG	Dagu	Zhuanghe city, Liaoning	800	9.1	170	2416	Temperate
EM	Emei Black	Mount Emei, Sichuan	1579	17.2	311	1100	Humid
HT	Hetian	Changting county, Fujian	1731	18.3	300	1785	Humid
LH	Luhua	Wenshang county, Shandong	1000	13.3	205	2285	Temperate
LS	Longsheng	Longsheng county, Guangxi	1500~2400	18.1	314	1244	Humid
NX1	Nixi	Zhongdian county, Yunnan	323	7.4~13.5	210	1987	Cold temperate
NX2							
NX3							
NY1	Niya	Minfeng county, Xinjiang	30.2	18.5	194	2842	Drought/Harsh
NY2							
NY3							
NY4							
NY5							
NY6							
NY7							
NY8							
NY9							
NY10							
NY11							
NY12							
NY13							
NY14							
NY15							
P1	Piao	Pu'er city, Yunnan	1284	18.5	318	1873~2206	Humid
P2							
P3							
QX	Qianxiang	Rongjiang county, Guizhou	1211	18.1	310	1313	Humid
QY	Qingyuan	Qingyuan city, Guangdong	1900	22	314	1662	Humid
TY	Taoyuan	Taoyuan county, Hunan	1506	17	279.5	1380	Humid
XJ	Xianju	Xianju county, Zhejiang	1560	17.2	310	2018	Humid
RJF1	Red jungle fowl	Yunnan/Hainan	1500–2000	_	_	_	Outgroup
RJF2							
RJF3							
RJF4							
RJF5							

a *The abbreviation of samples*.

b *The average precipitation, average annual temperature, frost-free period of the year, the annual sunshine duration from 2016 to 2020*.

To investigate putative genomic regions related to adaptation to hot arid and harsh environments in native chickens, we performed whole-genome resequencing of NYs together with other indigenous chickens belonging to 11 native chicken breeds that are widely distributed and reared in native humid and temperate areas across China. All Chinese indigenous chickens used in this study were dual-purpose types and has been suggested derived from the *G. gallus* spadiceus (Wang M.-S. et al., 2020). We combined several statistical approaches (*Fst* and θπ ratio, XP-CLR and Δ*AF*) to increase the statistical power to detect selection signatures in NYs. We focused on the main environmental factor (annual precipitation) to distinguish NYs and Others. Though other environmental factors do exists, but that was vague to distinguish NYs and Others from them. Our findings could broaden our understanding of thermal, drought, and harsh environment acclimation in chicken genomes and may serve as a valuable resource for developing new biotechnological tools to breed stress-tolerant chicken lines or breeds in the future. It is worth noting that the genomic differences between NYs and Others may not only be due to climate adaptation since other influence factors may exist due to the study design.

## Materials and Methods

### Sampling Information and Sequencing

The study was approved by the Biomedical Research Ethics Committee of Hunan Agricultural University. We performed the whole-genome resequencing of 15 unrelated NYs native to hot arid and harsh habitat. We also sequenced the whole genomes of 15 other indigenous chickens belonging to 11 native chicken breeds that are widely distributed and reared in native humid and temperate areas across China. Genomic DNA was extracted from venous blood of wing samples and muscle samples using the phenol-chloroform method. Sequencing libraries were generated using the Truseq Nano DNA HT Sample Preparation Kit (Illumina, USA). Whole genomes of 30 chickens were sequenced on the Illumina Hiseq 2500 platform for 150 bp paired-end reads on fast mode with two lines. Moreover, we downloaded the resequencing data of five Red Jungle Fowls (RJFs) (PRJNA241474) from NCBI.

### Quality Control, Reads Mapping, SNP Calling and Annotation

To ensure that reads were reliable and without artificial bias (low quality paired reads, which mainly resulted from base-calling duplicates and adapter contamination) in the following analyses, raw reads of fastq format were first processed through a series of quality control (QC) procedures using FastQC (Version 0.11.9). QC standards are as follows: (1) Removing reads with ≥10% unidentified nucleotides (N); (2) Removing reads with > 20% bases having Phred quality <5; (3) Removing reads with > 10 nt aligned to the adapter, allowing ≤ 10% mismatches; (4) Removing putative PCR duplicates generated by PCR amplification in the library construction process (read 1 and 2 of two paired-end reads that were completely identical).

The clean paired-end reads were aligned to the chicken reference genome (Galgal5) using Burrows-Wheeler Aligner (Version: 0.7.8) (settings: mem -t 8 -k 32–M -R). BAM alignment files were then generated using SAMtools (v.1.3) (Li et al., 2009). Additionally, we improved the alignment performance through the following steps: (1) filtering the alignment reads with mismatches ≤5 and mapping quality = 0, and (2) removing potential PCR duplication. If multiple read pairs had identical external coordinates, only the pair with the highest mapping quality was retained.

We further performed SNP calling on a population scale using the Genome Analysis Toolkit (GATK, version v3.6) with the HaplotypeCaller-based method. Before calling variants, the base quality scores were recalibrated using GATK, which provides empirically accurate base quality scores for each base in every read. After SNP calling, we applied variant quality recalibration to exclude potential false-positive variant calls. We used the command “Variant Filtration –filterExpression” with the following parameters: QD < 10.0; FS > 60.0; MQ < 40.0; ReadPosRankSum < −8.0; and -G_filter “GQ<20”. SNPs were annotated by ANNOVAR using Galgal5 database.

### Phylogenetic Tree and Population Structure

Considering that all the selected samples are from the *G. gallus*, the data are relatively similar and meet the conditions of superposition. An individual-based neighbor-joining tree was constructed based on the p-distance using TreeBestv1.9.2 software. We conducted principal component analysis (PCA) to evaluate genetic structure using GCTA software. The significance level of the eigenvector was determined using the Tracey-Widom test. The population genetic structure was examined via an expectation maximization algorithm, as implemented in the program FRAPPEv1.170. The number of assumed genetic clusters K ranged from 2 to 6, with 10,000 iterations for each run. Ancestral model-based clustering, with no prior knowledge on breed origins, was performed using ADMIXTURE 1.2.2 (Alexander et al., 2009) to investigate individual admixture proportions, for 1<k<10, where k is the number of expected subpopulations, and the best *k* = 2 was determined based on the cross-validation error for different numbers of ancestral genetic backgrounds.

### Selective Sweep Detection

We applied a sliding-window approach (40 kb windows sliding in 20 kb steps) according to Rubin et al. (2010) to quantify polymorphism levels (θπ, pairwise nucleotide variations as a measure of variability and genetic differentiation *F*_*ST*_ for NYs and Others, including LS, QY, HT, EM, XJ, TY, P1, P2, P3, QX which were breeds native to humid areas across China with average annual precipitation >1,200 mm). We calculated the θπ ratios based on the θπ values of the two populations and then log2-transformed the data. We considered the windows with the top 5% *F*_*ST*_ and log2 (θπ ratio) simultaneously as candidate outliers under strong selective sweeps in NYs. Furthermore, we estimated the XP-CLR statistic (Chen et al., 2010) between NY and Others. The threshold for candidate genes in the XP-CLR analyses was set to the top 1% percentile outliers. All outlier windows were assigned to corresponding SNPs and genes. Functional enrichment was performed for candidate genes using DAVID 6.8 (Huang et al., 2009).

### Absolute Allele Frequency Difference (*ΔAF*)

Allele frequency in a population is the number of individuals with a given genotype divided by the total number of individuals in the population (Brooker et al., 2011). The major of NY genotype was used as a reference, and the per genotype absolute allele frequency difference (Δ*AF*) between NYs and Others was then calculated with Perl script using the formula: Δ*AF* = abs (*AF*_*NYs*_ – *AF*_*Others*_).

## Results

### Mapping and SNP Calling

We sequenced 30 chickens (Supplementary Figure 1, Supplementary Table 1) to an average genome mapping rate of 99.05% with ~11.76-fold average depth (Supplementary Table 2). We identified 161,322 SNPs located within the coding region of the chicken genome, including 46,063 non-synonymous SNPs, 355 stop gain, 89 stop loss and 114,815 synonymous SNPs (Supplementary Table 3). Approximately 5.50 M SNPs were detected in each native chicken individual, and an average of 93.27% (range: 89.68–95.39%) of the SNPs were validated in the chicken dbSNP (Build 151) database (Supplementary Table 4), indicating the high reliability of the called SNP variations in this study.

### Phylogeny and Population Structure Analysis

**Figure 1:**
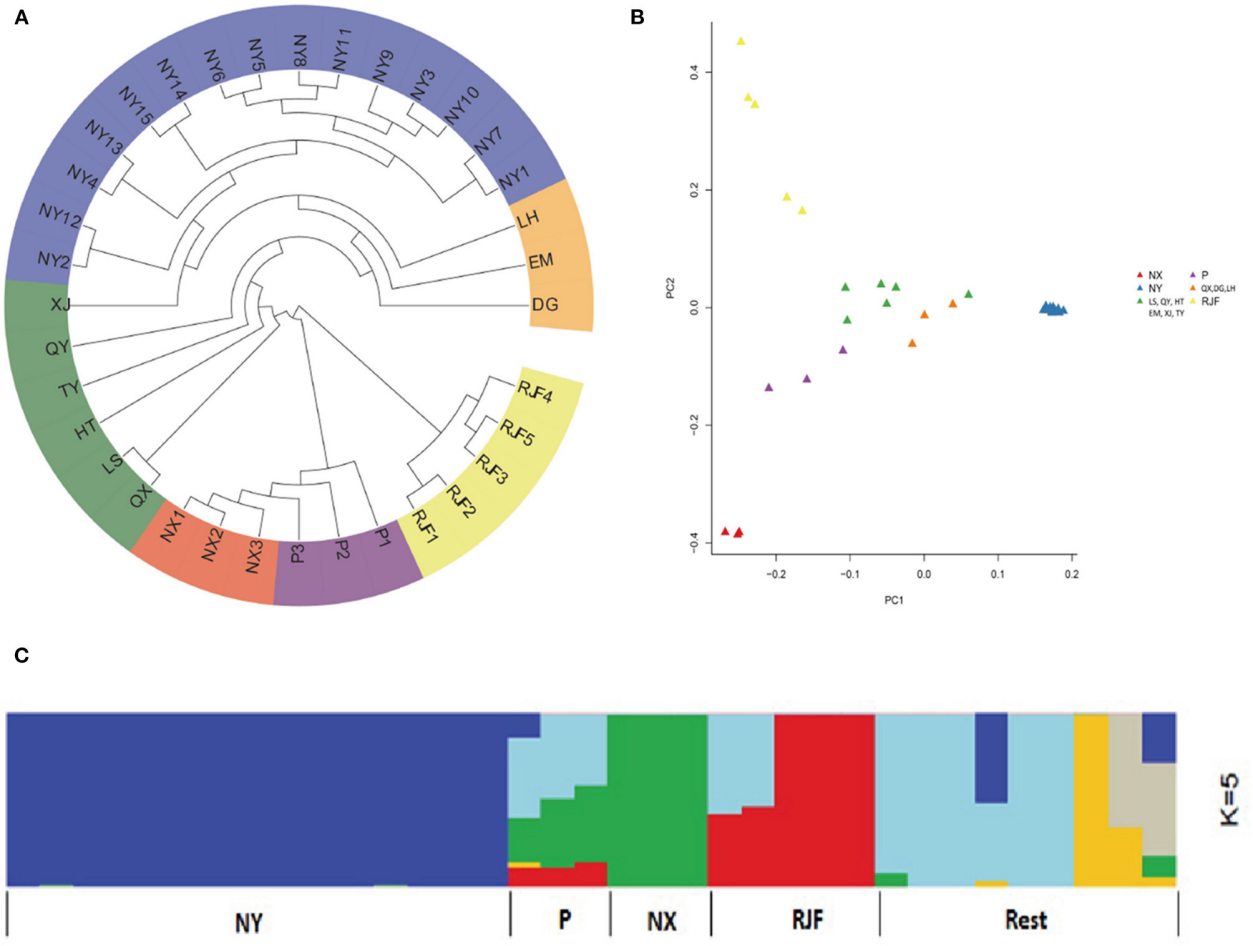
Population genetics analyses of 35 chickens. **(A)** Neighbor-joining tree constructed using p-distances between individuals. **(B)** Principal component analysis (PCA) of 35 chickens. **(C)** Genetic structure of chicken breeds. The length of each colored segment represents the proportion of the individual's genome from ancestral populations (*k* = 5). The population names are at the bottom of the figure. Dagu (DG), Emei Black (EM), Hetian (HT), Luhua (LH), Longsheng (LS), Nixi (NX), Niya (NY), Piao (P), Qianxiang (QX), Qingyuan (QY), Taoyuan (TY), Xianju (XJ), and Red jungle fowl (RJF).

To investigate the genetic relationships between NYs and other native chicken breeds, we constructed a phylogenetic tree using the neighbor-joining method. From the phylogenic tree, the RJF formed a root clade that was separated from other domesticated chicken breeds (Figure 1A). Six individuals belonging to two chicken breeds (NX and P) first separated and NYs with the rest chickens formed a subcluster. The PCA analysis distinguished NY from other chickens and RJFs (Figure 1B). In particular, the first eigenvector PC1 separated NYs from others, explaining 8.24% of the overall variation, and the second eigenvector PC2 distinguished NXs and RJFs, explaining 5.15% of the overall variation (Supplementary Table 5). We further performed population admixture analysis to examine the possible population ancestry and structure of NYs. When the pre-defined genetic cluster K was set to 2, we found that other native breeds together with RJFs were separated from NY chickens. When K was set to 3, other breeds (P, NX and rest samples) were further separated from RJF. When K was set to 4, RJFs were divided into two lineages, as reported previously (Wang et al., 2015), and more additional lineages of other breeds were found, indicating the possible admixture among native breeds. Notably, however, at all settings of K (2–5) in this study, NYs were remain separated from other local breeds, implying possible isolation with other indigenous chicken breeds during the breeding history of NY (Figure 1C).

### Positive Selective Signatures in Niya Chicken

**Figure 2:**
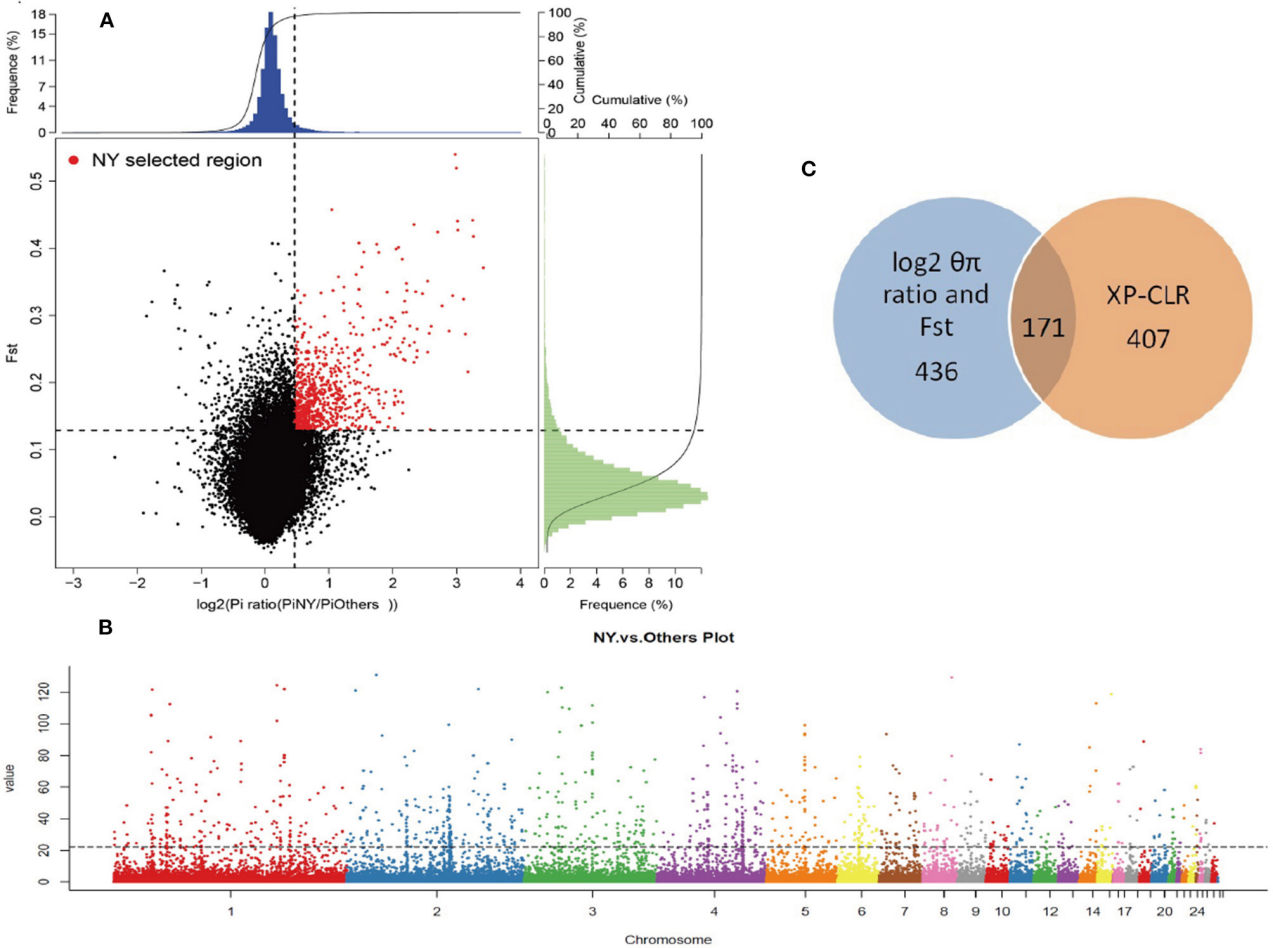
Genomic regions with strong selective sweep signals in NYs. **(A)** Distribution of log2 (θπ ratio [θπ, Others / θπ, NYs]) (top 5% outliers, log2 [θπ ratio] > 0.46) and *F*_*ST*_ values (top 5% outliers, *F*_*ST*_ > 0.13), which are calculated in 40 kb windows sliding in 20 kb steps. The selected regions for NYs were indicated by red dots. **(B)** The Manhattan plot shown candidate genes positively selected in XP-CLR method between NYs and Others. The dotted line indicates the top 1% threshold. The data points above the straight line (corresponding to the top 1% of empirical values) are genomic regions under selection in NYs. **(C)** A total of 171 candidate genes in NYs were identified by combining three methods (including log2 [θπ ratio], *F*_*ST*_ and XP-CLR) simultaneously and are shown using Venn diagram components.

To reliably detect the genomic footprints left by selection pressures related to adaptation to hot, dry, and harsh environments in NYs and reduce the impact of genetic divergence that results from the domestication process, we measured pairwise comparisons of genome-wide variations and compared NYs with a domesticated diverse local chicken panel (Others) (Supplementary Table 6). We used an empirical procedure and selected regions simultaneously with significantly higher log2 (θπ ratio [θπ, Others/θπ, NYs]) (top 5% outliers, log2 [θπ ratio] > 0.46) and *F*_*ST*_ values (top 5% outliers, *F*_*ST*_ > 0.13) as candidate regions with strong selective sweep signals along the genome (except for the sex chromosome) (Figure 2A). We identified a total of 32.4 Mb genomic regions (3.07% of the genome, containing 436 genes, 24 chromosomes, the average length is 67.2 kb) with strong selective sweep signals in NYs (Supplementary Tables 7, 8).

We also ran a composite likelihood ratio test (XP-CLR) between the two populations (NYs and Others) to search for the selective sweeps. We divided the whole genome area into non-overlapping windows of 10 kb. The genomic windows with XP-CLR scores greater than a threshold of 22.85 (top 1% outliers) were putative selected regions. We identified a total of 8.67 Mb genomic regions (0.81% of the genome, containing 407 genes, 26 chromosomes, the average length is 24.9 kb) with strong selective sweep signals in NYs (Figure 2B, Supplementary Tables 9, 10). The combination of different statistical analysis results would provide more powerful information than a single test result. Thus, we obtained 672 putative genes (Supplementary Table 11) in the union of log2 θπ ratio, *F*_*ST*_ and XP-CLR after removed the duplicates and 171 genes intersected with three statistics (Figure 2C, Supplementary Table 12). These overlapped putative genes were significantly functionally enriched in p53 signaling, metabolic pathway and 14 GO terms (corrected *P*-value < 0.05) which may play important roles in hot arid adaptation (such as the *NRP1* and *PARK2*) in NYs (Table 2). The functional analysis of candidate genes using David for the gene list results from each of the statistical analyses was showed in Supplementary Tables 13, 14.

**Table 2:** Enriched genes associated with adaptation to hot arid and harsh environment in NYs (top list, corrected *P*-value ≤ 0.05).

**Category**	**Term description**	**Involved gene number**	**Corrected *P*-value**
GO:0015171	Amino acid transmembrane transporter activity	4	7.61E-03
KEGG: gga04115	P53 signaling pathway	6	1.08E-02
GO:0000151	Ubiquitin ligase complex	6	1.38E-02
GO:0042383	Sarcolemma	5	1.40E-02
GO:0003334	Keratinocyte development	3	1.46E-02
GO:0055003	Cardiac myofibril assembly	3	1.46E-02
GO:0006364	rRNA processing	5	1.69E-02
GO:0042104	Positive regulation of activated T cell proliferation	3	1.91E-02
GO:0006312	Mitotic recombination	3	2.42E-02
GO:0016887	ATPase activity	7	2.66E-02
GO:0006893	Golgi to plasma membrane transport	3	2.97E-02
GO:0005578	Proteinaceous extracellular matrix	9	3.31E-02
GO:0004860	Protein kinase inhibitor activity	4	4.60E-02
GO:0032012	Regulation of ARF protein signal transduction	3	4.87E-02
GO:0007409	Axonogenesis	5	4.90E-02
KEGG: gga01100	Metabolic pathways	32	4.97E-02

## Discussion

We used combined statistical analysis to search for putative genes that might be related to the adaptation of hot arid and harsh environment in NYs. We obtained a list of intersected candidate genes with three statistical analyses (including 123 regions of 21 chromosomes with the average length of 54.4kb) (Supplementary Table 10) involved in different molecular processes and pathways implied complex genetic mechanisms of adaptation of native chickens to hot arid and harsh environments.

The living environment of NYs is characterized by huge diurnal ambient temperature differences caused by intense solar radiation, resulting in high temperatures during the day. Chickens use a variety of methods to maintain core body temperature and internal homeostasis (Lara and Rostagno, 2013), including increased radiation, convective, and evaporative heat loss through the diastolic blood vessels (Mutaf et al., 2009). By reducing blood flow bypassing internal organs, more blood flows to naked surface tissues, such as combs (Wolfenson et al., 1981) and bare feet (Hillman et al., 1982), to dissipate excessive heat. Additionally, as a true panting animal, chickens can expel internal heat by evaporating moisture through the lungs and air sacs, which are richer in blood vessels (Collier and Gebremedhin, 2015).

In NYs, we found several genes that were related to circulatory and cardiovascular system development, blood vessel development, and heart development. For example, BVES is known as a caveolae-associated protein important for the preservation of caveolae structural and functional integrity and heart protection (Alcalay et al., 2013). It also plays a role in the regulation of heart rate (Froese et al., 2012), response to ischemia (Alcalay et al., 2013), and heart development (Torlopp et al., 2006). SMYD1 is essential for cardiomyocyte differentiation and cardiac morphogenesis (Phan et al., 2005) and involved in angiogenesis (Ye et al., 2016). PDGFRA plays the role of the receptor and the ligands in chicken cardiac development (Bax et al., 2009). IL18 is a kind of cytokine with multiple proinflammatory effects and is suggested to be a novel angiogenic mediator (Park et al., 2001). *NRP1* encodes neuropilin 1, a type of VEGF receptor, and has been well-studied with known functions in angiogenesis (Gelfand et al., 2014), cardiovascular system development (Ruhrberg et al., 2009), the formation of certain neural circuits (Schwarz et al., 2008), and the development in other organs other than nervous system organs (Ng et al., 2001). And selective signals of *IL18* and *NRP1* were just consistent with the adaptation studies in East-African vs. North-African chickens (Elbeltagy et al., 2019). Interestingly, in NY populations, we found high Δ*AF* (Δ*AF* > 0.7) only presented in 18 intronic SNPs of *CORIN* compared with other domestic chickens. CORIN is a heart hormone that regulates blood pressure and volume in humans (Chen et al., 2015) and could be functionally important for the maintenance of the proper volume of blood fluids in NYs due to adapt to hot arid environments. Therefore, we assumed that selected genes involved in the circulatory system and vasculature development reflect the crucial role of thermoregulation in adaptation to hot arid environments in NYs.

In the selected genes, we identified genes related to central nervous system development including dendrite development, neuron migration, neuron differentiation, and brain development. For example, SIM2 is a major regulator of neurogenesis (Moffett et al., 1997). *NALCN* regulates sodium leakage conductance responsible for the neuronal background. Many studies in humans and mice have shown alterations, retardation, and deleterious effects in the central nervous system due to adaptation to thermal environments (Ahmed, 2005). Therefore, we supposed that the selective signals of genes related to central nervous system development in NYs may be the result of adaptation to the hot arid environment.

Apoptosis refers to a stable and highly regulated cell death process controlled by multiple genes and has functions to maintain the stability of the internal environment in living organisms. Also, it permits adaptation to the existing environment (Elmore, 2007). We found abundant apoptosis-related genes in the selective regions. For example, *CLPTM1L* encodes a membrane protein associated with apoptosis induced by cisplatin. In particular, we found an abundance of genes with functions related to cell differentiation and apoptosis in neural cells, such as *APP, CRADD* and *PARK2*. And studies of Elbeltagy et al. (2002) reported that *PAPK7* were located at the ROH region of North African Chicken populations (Elbeltagy et al., 2019). Studies have shown the heat-induced apoptosis mainly manifests as central nervous system damage and can cause cellular injury in the liver, heart, and kidneys of humans and mice (Edwards et al., 1997; Bongiovanni et al., 1999). Hence, we speculated that the set of genes involved in apoptosis, particularly induction of neural cell death, may provide evidence of the adaptation of NYs to the hot arid environment.

The living environments of NYs are highly stressful (e.g., thermal stress, arid and sandy wind, and solar radiation). To maintain homeostasis and thermoregulation, chickens can use various physiological activities to respond to such external stimuli. We found that selected genes were associated with response to hormones, endogenous stimuli, and external stimuli. Notably, the *AHR* gene encodes aryl hydrocarbon receptor, a ligand-activated transcription factor that is highly conserved in different species and is involved in eliminating various environmental toxins, mediating immune defenses, and regulating metabolism and cellular stress (Moura-Alves et al., 2014). ESRRG is a steroid hormone receptor and plays a critical role in thermogenesis (Ahmadian et al., 2018). Moreover, *FAS* and *IL18* play important roles in immune and inflammatory responses (Wawrocki et al., 2016; Guegan and Legembre, 2018). Also, UBE4B belongs to E3 ubiquitin ligases and allows cells to respond to a variety of stimuli to keep their internal environment stable (Karve and Cheema, 2011) and also involved in DNA damage repair (Ackermann et al., 2016). And two E3 ligases were just identified as the selection signatures among Egyptian chicken populations compared with that Brazilian and Sri Lankan (Walugembe et al., 2019). The strong selected signals in these genes indicate that NYs may have better disease resistance and stronger ability to survive in the harsh environment than other chickens evaluated in this study.

Chickens seem to be more sensitive to environmental stressors (particularly heat stress) (Lara and Rostagno, 2013). When broilers encounter chronic heat stress, physiological performance is altered, including stimulation of lipid accumulation, reducing fat decomposition, and enhancing the metabolism of amino acids (Geraert et al., 1996). Among the selected genes, FABP1, a fatty acid binding protein, has several functions including the mediation of catabolism or anabolism of lipid metabolic pathways, the preservation of intracellular fatty acid level and the regulation of FA-responsive genes transcriptions (Wang et al., 2019). *FABP1* may be important for maintaining lipid metabolism and normal growth rates in NYs due to acclimation to the hot arid environment. By comparing the intramuscular fat (IMF) contents in three tissues (the cardiac, chest and leg muscles) between NY and three-yellow (TYC) chicken (a major meat-type broiler raised in China), NY showed significantly increased IMF contents than TYC (Wang et al., 2016).

To the best of our knowledge, our findings revealed the complex genetic mechanisms of adaptation to hot arid and harsh environments of native chickens in China for the first time. Although functional studies are further needed to confirm our findings, our work could improve our understanding of thermal, drought, and harsh environment acclimation in chicken genomes.

## Data Availability Statement

Whole-genome resequencing data for 30 unrelated chicken individuals have been deposited in the NCBI Sequence Read Archive (SRA) under bioproject number PRJNA344300 and PRJNA553299. Previously published genome sequences from five Red Jungle Fowls (bioproject number PRJNA241474) were downloaded from the NCBI database.

## Ethics Statement

The animal study was reviewed and approved by Biomedical Research Ethics Committee of Hunan Agricultural University.

## Author Contributions

JG and SL designed the project. JG drafted the manuscript with inputs from all authors. JG, QL, CL, and SL wrote and revised the manuscript. JG collected the chicken samples. JG, QL, CL, and SL analyzed the data. All the authors revised and approved the manuscript.

## Conflict of Interest

QL and CL were employed by the company Novogene Bioinformatics Institute. SL was employed by the company Maxun Biotechnology Institute. The remaining author declares that the research was conducted in the absence of any commercial or financial relationships that could be construed as a potential conflict of interest.
