# Supplementary material for: Genomic Analyses Reveal Adaptation to Hot Arid and Harsh Environments in Native Chickens of China
Source: Front Genet. 2020 Dec 21;11:582355. doi: 10.3389/fgene.2020.582355 (PMC7793703; doi:10.3389/fgene.2020.582355)
Supplement: Supplementary file 1 [file Data_Sheet_1.PDF]

Genomic analyses reveal adaptation to hot arid and harsh environments in native chickens

Jingjing Gu[1,3,5†*], Qiqi Liang[†4], Can Liu[†4] and Sheng Li[2*]

[1]College of Animal Science and Technology, Hunan Agricultural University, Changsha, China. [2]Maxun Biotechnology Institute, Changsha, China. [3]Hunan Provincial Key Laboratory for Genetic Improvement of Domestic Animal, Changsha, China. [4]Novogene Bioinformatics Institute, Beijing, China. [5]Hunan Engineering Research Center of Poultry Production Safety, Changsha, China.

†These authors have contributed equally to this work.

*Correspondence:

Jingjing Gu hnaugu@163.com

Sheng Li maxunls@aliyun.com

Supplementary Information

Supplementary Figures	Page
Supplementary Figure 1	3
Supplementary Figure 2	4

Supplementary Tables	Page
Supplementary Table S1	5
Supplementary Table S2	6
Supplementary Table S3	7
Supplementary Table S4	8-9
Supplementary Table S5	10
Supplementary Table S6	10
Supplementary Table S7	10-20
Supplementary Table S8	21-30
Supplementary Table S9	31-40
Supplementary Table S10	40-49
Supplementary Table S11	49-64
Supplementary Table S12	65-68
Supplementary Table S13	69
Supplementary Table S14	69
Supplementary Table S15	70

Supplementary Figures

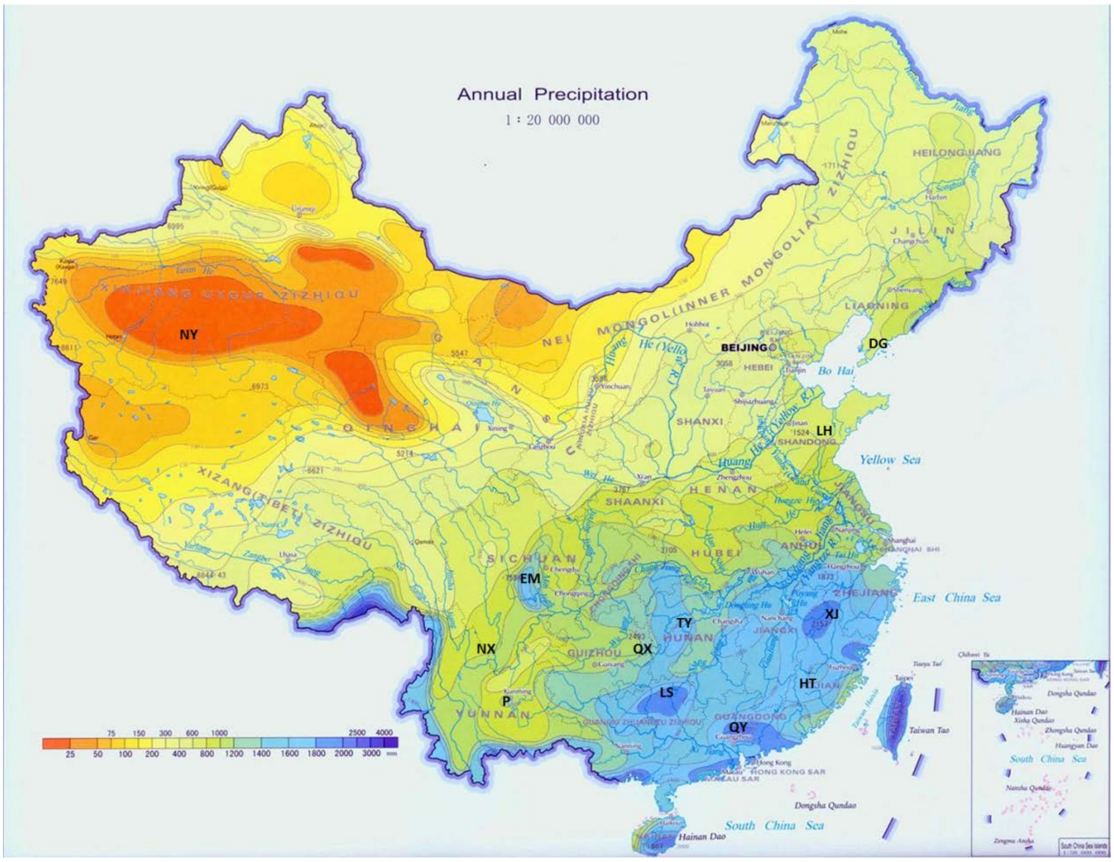

Supplementary Figure 1 Diagram of average annual precipitation in China with the geographic distribution of the chicken samples. The map was produced using Google Maps. Sample abbreviations: Dagu (DG), Emei Black (EM), Hetian (HT), Luhua (LH), Longsheng (LS), Nixi (NX), Niya (NY), Piao (P), Qianxiang (QX), Qingyuan (QY), Taoyuan (TY), Xianju (XJ), and Red jungle fowl (RJF).

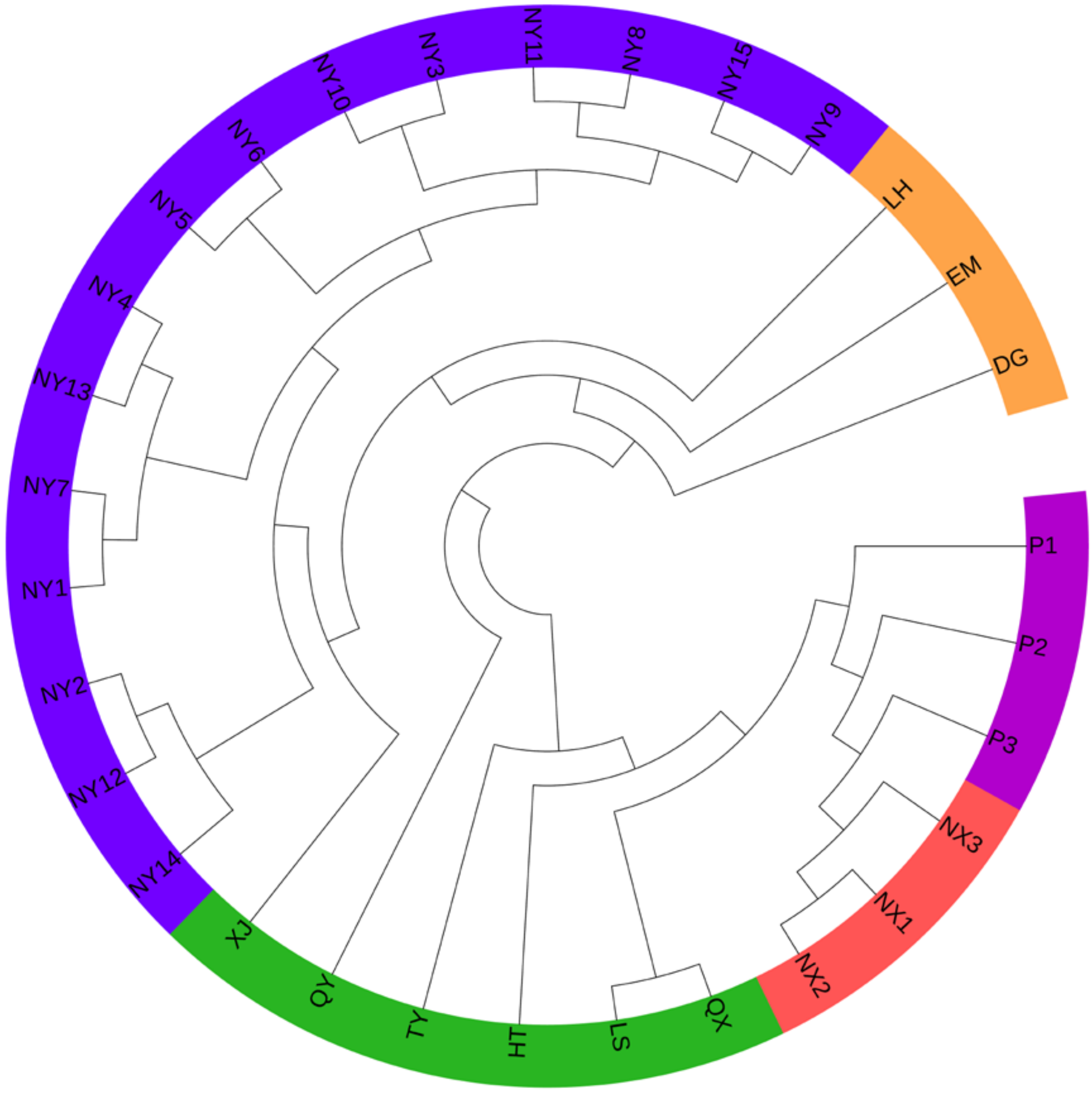

Supplementary Figure 2 Neighbor-joining tree constructed using p-distances between chicken individuals without Red jungle fowl (RGF). Abbreviation: Dagu (DG), Emei Black (EM), Hetian (HT), Luhua (LH), Longsheng (LS), Nixi (NX), Niya (NY), Piao (P), Qianxiang (QX), Qingyuan (QY), Taoyuan (TY) and Xianju (XJ).

Supplementary Table S1 Geographic location distribution of the chicken population used in this study.

Individual Name[1]	Breeds	Geographic locations	Annual precipitation (mm)[2]	Environment Characteristics
DG	Dagu	Zhuanghe City, Liaoning	800	Temperate
EM	Emei Black	Mount Emei, Sichuan	1579	Humid
HT	Hetian	Changting County, Fujian	1731	Humid
LH	Luhua	Wenshang County, Shandong	1000	Temperate
LS	Longsheng	Longsheng County, Guangxi	1500-2400	Humid
NX1				
NX2	Nixi	Zhongdian County, Yunnan	323	Cold temperate
NX3				
NY1				
NY2				
NY3				
NY4				
NY5				
NY6				
NY7				
NY8	Niya	Minfeng County, Xinjiang	30.2	Drought/Harsh
NY9				
NY10				
NY11				
NY12				
NY13				
NY14				
NY15				
P1				
P2	Piao	Pu'er City, Yunnan	1284	Humid
P3				
QX	Qianxiang	Rongjiang County, Guizhou	1211	Humid
QY	Qingyuan	Qingyuan City, Guangdong	1900	Humid
TY	Taoyuan	Taoyuan County, Hunan	1506	Humid
XJ	Xianju	Xianju County, Zhejiang	1560	Humid
RJF1				
RJF2				
RJF3	Red jungle fowl	Yunnan / Hainan	1500-2000	Outgroup
RJF4				
RJF5				

Notes: [1]The abbreviation of samples; [2] The average precipitation from 2016 to 2020.

Supplementary Table S2 Summary and mapping statistics of sampled chicken populations

Individual ID	Raw Base (Gb)	High quality data				Average depth	Coverage at least 1X	Coverage at least 4X
		Base (Gb)	Q20 (%)	Q30 (%)	Mapping Rate (%)			
DG	12.52	12.05	95.09	90.92	98.99	10.42	91.15	83.09
EM	12.86	12.63	95.02	90.81	99.30	11.01	91.34	83.45
HT	10.42	10.21	94.52	89.99	99.19	9.02	90.74	79.89
LH	12.78	12.54	94.56	90.04	99.20	10.97	91.21	82.56
LS	10.94	10.71	94.74	90.41	99.32	9.50	90.63	80.71
NX1	11.26	11.04	93.85	88.92	99.08	9.70	91.01	81.27
NX2	9.96	9.78	95.19	91.14	99.36	8.65	90.66	79.92
NX3	14.52	13.57	94.97	90.67	98.97	11.94	90.95	82.21
NY1	12.14	11.87	91.92	86	99.36	10.49	90.21	84.29
NY2	11	10.76	95.08	90.88	99.34	9.54	90.17	82.08
NY3	10.96	10.69	91.21	85.27	99.33	9.51	90.04	81.96
NY4	11.28	11.20	94.28	87.45	98.84	8.99	90.09	82.78
NY5	11.12	11.06	94.06	87.1	98.83	8.79	90.82	81.43
NY6	21.80	21.72	93.66	86.36	98.79	17.22	91.09	88.44
NY7	10.93	10.90	93.47	86.09	98.59	8.82	90.65	81.50
NY8	11.55	11.48	94.44	87.95	98.60	9.04	90.93	82.04
NY9	12.25	12.19	93.73	86.61	98.48	9.61	91.05	83.36
NY10	12.31	12.23	96.35	91.94	98.54	9.72	91.08	83.25
NY11	12.72	12.68	96.73	92.62	98.69	10.09	91.11	84.20
NY12	14.06	14.01	96.8	92.71	98.21	11.07	91.23	85.43
NY13	15.30	15.26	96.42	92	98.67	11.96	91.52	86.47
NY14	13.83	13.78	97.05	93.28	98.73	10.98	91.21	85.30
NY15	13.66	13.61	96.85	92.87	98.73	10.78	91.32	85.23
P1	11.45	11.17	91.08	85.06	99.38	9.82	90.91	82.20
P2	11.89	11.68	94.89	90.6	99.31	10.28	90.88	83.18
P3	12.45	11.92	95.06	90.83	99.14	10.51	90.84	82.84
QX	12.69	12.28	94.95	90.62	99.41	10.85	90.98	82.48
QY	11.22	10.99	93.76	88.78	99.06	9.62	91.15	81.19
TY	12.08	11.7	94.61	90.12	99.18	10.33	90.81	82.54
XJ	11.8	11.6	95.53	91.59	99.45	10.27	90.86	83.08
RJF1	17.34	15.57	92.66	81.14	98.60	13.36	92.08	86.86
RJF2	28.6	28.01	93.29	87.61	99.46	23.04	93.53	90.51
RJF3	18.08	16.75	94.31	84.37	99.51	14.76	90.41	85.51
RJF4	21.71	20.5	94.95	85.76	99.47	17.86	91.00	87.68
RJF5	40.23	38.7	94.24	87.39	99.53	33.20	91.73	89.48

Supplementary Table S3 Summary of SNP calling on a population-scale

Category		Number of SNP	Not in Chicken dbSNP build 151	In Chicken dbSNP build 151
Upstream[1]		166,633	23,162	139,763
Intronic		4373061	313,726	4,036,002
Splicing[2]		331	45	273
Exonic	Non-synonymous	46,063	6,177	37,790
	Synonymous	114,815	11,211	100,765
	Stop gain	355	47	293
	Stop loss	89	10	73
	Non-synonymous/Synonymous	40.12%	55.10%	37.50%
Downstream[3]		144,436	13,443	127,327
Upstream/Downstream[4]		7,667	1,393	6,004
Intergenic		6,069,169	466,997	5,527,406
Total		10,922,619	836,211	9,975,696

Notes: [1]1 Kb before ATG; [2]Mutation at Splicing site (2bp near exon/intron boundary in introns); [3]Downstream: 1 Kb after TGA; [4] Upstream/Downstream: a region of 1 Kb Upstream of a gene, but at the same time it is also in the Downstream of another gene.

Supplementary Table S4 Summary information of detection SNPs identified based with Gallus gallus dbSNP database

Individual ID	Total SNPs	Number of Heterozygous	Heterozygous rate of Genome (‰)	SNP in Placed Chromosome	Gallus gallus dbSNP 151		Identified Rate in dbSNP151	Validated Rate in dbSNP151
					Validated	No. Validated		
NY1	5,727,429	3,375,623	2.765	5,638,052	5450949	187103	26.28%	95.17%
NY2	5,433,044	3,224,251	2.641	5,344,887	5158909	185978	24.87%	94.95%
NY3	5,486,571	3,177,175	2.603	5,398,491	5223534	174957	25.19%	95.21%
NY4	5,202,333	2,961,856	2.427	5,121,399	4962445	158954	23.93%	95.39%
NY5	5,078,395	2,850,196	2.335	4,993,706	4823047	170659	23.25%	94.97%
NY6	6,123,510	3,689,304	3.022	6,010,810	5802628	208182	27.98%	94.76%
NY7	5,167,465	2,933,302	2.403	5,086,952	4920167	166785	23.72%	95.21%
NY8	5,144,308	2,905,306	2.38	5,057,782	4890909	166873	23.58%	95.07%
NY9	5,326,086	3,040,199	2.491	5,235,345	5055180	180165	24.37%	94.91%
NY10	5,285,429	3,025,058	2.478	5,190,055	5014106	175949	24.18%	94.87%
NY11	5,442,503	3,155,364	2.585	5,343,784	5152116	191668	24.84%	94.66%
NY12	5,602,140	3,261,603	2.672	5,502,078	5304613	197465	25.58%	94.69%
NY13	5,681,268	3,314,020	2.715	5,574,590	5383613	190977	25.96%	94.76%
NY14	5,557,607	3,250,791	2.663	5,455,068	5262168	192900	25.37%	94.68%
NY15	5,554,205	3,249,629	2.662	5,455,372	5263118	192254	25.38%	94.76%
DG	5,574,057	3,159,440	2.588	5,474,217	5191570	282647	25.03%	93.14%
EM	5,564,673	3,129,518	2.563	5,459,893	5174708	285185	24.95%	92.99%
HT	5,394,392	3,092,333	2.533	5,303,319	4930835	372484	23.77%	91.41%
LH	5,431,41	2,953,162	2.419	5,319,792	5078241	241551	24.48%	93.50%

	2							
LS	5,497,503	3,168,447	2.595	5,419,049	4988470	430579	24.05%	90.74%
NX1	5,423,274	2,872,968	2.353	5,323,033	4878017	445016	23.52%	89.95%
NX2	5,190,349	2,728,202	2.235	5,107,638	4702117	405521	22.67%	90.59%
NX3	5,576,776	3,011,528	2.466	5,472,175	5028443	443732	24.24%	90.17%
P1	5,711,618	3,454,059	2.829	5,625,011	5235444	389567	25.24%	91.66%
P2	5,554,139	3,083,328	2.525	5,472,391	5054460	417931	24.37%	91.00%
P3	5,708,617	3,333,178	2.73	5,619,682	5119499	500183	24.68%	89.68%
QX	5,734,922	3,450,617	2.826	5,636,359	5213379	422980	25.14%	90.91%
QY	5,467,451	3,193,409	2.616	5,362,509	5019459	343050	24.20%	91.81%
TY	5,697,625	3,355,018	2.748	5,603,103	5240878	362225	25.27%	91.98%
XJ	5,589,100	3,270,611	2.679	5,511,858	5278656	233202	25.45%	94.45%
RJF1	5,971,562	3,494,250	2.862	5,883,131	5328963	554168	25.69%	89.24%
RJF2	6,465,280	3,771,347	3.088	6,349,802	5694260	655542	27.45%	88.07%
RJF3	6,635,871	4,080,363	3.341	6,567,316	5486359	1080957	26.45%	82.68%
RJF4	6,504,788	3,543,214	2.901	6,416,693	5479845	936848	26.42%	84.24%
RJF5	7,419,192	4,806,935	3.935	7,294,109	6046906	1247203	29.16%	81.50%

Supplementary Table S5 The first to five eigenvalues from PCA analysis of four chicken population

Number	Eigenvalue	Percentage %
1	3.07534	8.24
2	1.91941	5.15
3	1.51532	4.06
4	1.3862	3.72
5	1.21943	3.27

Supplementary Table S6 Diversity levels of three chicken populations

Population	Sample size	$\theta\pi(10^{-3})$	$\theta\omega(10^{-3})$
NY	15	3.2115	2.5438
Others	10	3.4065	2.9082
RJF	5	3.3658	3.0874

Note: Others panel includes those samples: LS, QY, HT, EM, XJ, TY, P1, P2, P3, QX

Supplementary Table S7 Genomic regions with strong selective sweep signals in NYs defined by significantly higher log2 (θπ ratio [θπ, Others / θπ, NYs]) (top 5% outliers, log2 [θπ ratio] > 0.46) and F_{ST} values (top 5% outliers, F_{ST} > 0.13) .

Chromosome	Location Start (bp)	Location End (bp)	Selective Sweep Length (kb)	log2 (θπ ratio [θπ, Others / θπ, NYs])	Fst value	Gene ID
1	10320001	10360000	40.0	0.46	0.14	ENSGALG00000008486
1	11000001	11040000	40.0	0.55	0.15	ENSGALG00000040444
1	17240001	17300000	60.0	0.74	0.16	ENSGALG00000023584
1	30960001	31080000	120.0	0.50	0.14	ENSGALG00000008497, ENSGALG00000009698, ENSGALG00000043800, ENSGALG00000025619
1	32700001	32740000	40.0	0.48	0.13	ENSGALG00000019361
1	34060001	34100000	40.0	0.72	0.17	ENSGALG00000026661
1	39240001	39300000	60.0	0.53	0.16	ENSGALG00000041094
1	39860001	39920000	60.0	1.10	0.26	ENSGALG00000010935
1	43920001	43980000	60.0	0.48	0.24	ENSGALG00000011269, ENSGALG00000011270,

						ENSGALG00000011271
1	44780001	44820000	40.0	0.51	0.16	ENSGALG00000011285
1	44880001	44940000	60.0	0.74	0.19	ENSGALG00000011297, ENSGALG00000011295
1	49080001	49120000	40.0	0.61	0.27	ENSGALG00000011854
1	55300001	55360000	60.0	0.67	0.21	ENSGALG00000012755
1	55500001	55540000	40.0	0.57	0.13	ENSGALG00000012760, ENSGALG00000012761
1	55900001	55940000	40.0	0.69	0.13	ENSGALG00000012791
1	68760001	68820000	60.0	0.82	0.15	ENSGALG00000014178
1	71840001	71880000	40.0	0.61	0.17	ENSGALG00000031503
1	72340001	72400000	60.0	0.47	0.14	ENSGALG00000036730, ENSGALG00000026372
1	73240001	73300000	60.0	0.83	0.15	ENSGALG00000017283
1	73400001	73660000	260.0	0.68	0.14	ENSGALG00000033533, ENSGALG00000017282, ENSGALG00000040681, ENSGALG00000036149, ENSGALG00000029785, ENSGALG00000029785, ENSGALG00000033533, ENSGALG00000039534, ENSGALG00000041941, ENSGALG00000036222, ENSGALG00000017280
1	74240001	74400000	160.0	0.49	0.23	ENSGALG00000017271, ENSGALG00000017272
1	75760001	75860000	100.0	0.68	0.23	ENSGALG00000014329, ENSGALG00000037656, ENSGALG00000042678
1	76420001	76460000	40.0	1.37	0.14	ENSGALG00000011930, ENSGALG00000040898, ENSGALG00000026901
1	84600001	84660000	60.0	0.63	0.15	ENSGALG00000015267
1	102680001	102720000	40.0	0.49	0.16	ENSGALG00000015770
1	106760001	106800000	40.0	0.57	0.14	ENSGALG00000016044
1	107260001	107300000	40.0	0.53	0.17	ENSGALG00000016054
1	119060001	119180000	120.0	0.50	0.18	ENSGALG00000016382, ENSGALG00000019157, ENSGALG00000033942, ENSGALG00000016391, ENSGALG00000016388
1	137440001	137500000	60.0	1.49	0.20	ENSGALG00000016828, ENSGALG00000046372,

						ENSGALG00000037697
1	142760001	142800000	40.0	0.52	0.14	ENSGALG00000016861, ENSGALG00000016860, ENSGALG00000016862, ENSGALG00000016863
1	142780001	142820000	40.0	0.65	0.16	ENSGALG00000016862, ENSGALG00000016861, ENSGALG00000016863
1	143640001	143700000	60.0	0.68	0.26	ENSGALG00000016868
1	147800001	147920000	120.0	0.81	0.15	ENSGALG00000016902
1	148360001	148460000	100.0	2.15	0.17	ENSGALG00000038439, ENSGALG00000027893
1	150280001	150360000	80.0	2.58	0.13	ENSGALG00000032339, ENSGALG00000035126, ENSGALG00000039004
1	160600001	160840000	240.0	1.20	0.15	ENSGALG00000045240, ENSGALG00000045907
1	161740001	161780000	40.0	0.86	0.17	ENSGALG00000030557, ENSGALG00000040645
1	177080001	177140000	60.0	0.55	0.15	ENSGALG00000017111, ENSGALG00000017112
1	186280001	186320000	40.0	1.10	0.16	ENSGALG00000017228
2	300001	340000	40.0	0.68	0.16	ENSGALG00000036190, ENSGALG00000042836, ENSGALG00000045908
2	13220001	13260000	40.0	0.67	0.17	ENSGALG00000007125
2	13880001	14020000	140.0	0.47	0.14	ENSGALG00000007140
2	20080001	20120000	40.0	0.67	0.18	ENSGALG00000040891
2	24500001	24560000	60.0	0.61	0.18	ENSGALG00000027620
2	26360001	26440000	80.0	0.87	0.14	ENSGALG00000010718
2	28820001	28900000	80.0	0.59	0.15	ENSGALG00000010836
2	29040001	29080000	40.0	0.49	0.25	ENSGALG00000010840
2	43640001	43680000	40.0	0.66	0.18	ENSGALG00000036110
2	48500001	48540000	40.0	0.72	0.16	ENSGALG00000039935
2	49680001	49920000	240.0	1.31	0.22	ENSGALG00000031713
2	50140001	50220000	80.0	0.56	0.21	ENSGALG00000031758, ENSGALG00000031294, ENSGALG00000035959
2	50720001	50780000	60.0	1.78	0.28	ENSGALG00000034616
2	50840001	50900000	60.0	2.19	0.32	ENSGALG00000040266
2	56900001	56960000	60.0	0.91	0.16	ENSGALG00000039442, ENSGALG00000031323
2	62160001	62200000	40.0	0.49	0.16	ENSGALG00000012715
2	62920001	62960000	40.0	0.61	0.14	ENSGALG00000012739

2	63080001	63120000	40.0	0.58	0.18	ENSGALG00000032683
2	73320001	73360000	40.0	0.93	0.16	ENSGALG00000012941
2	76400001	76440000	40.0	1.10	0.14	ENSGALG00000012964
2	81400001	81460000	60.0	0.54	0.20	ENSGALG00000013102
2	85960001	86120000	160.0	0.74	0.20	ENSGALG00000019527, ENSGALG00000013180, ENSGALG00000013183, ENSGALG00000013187, ENSGALG00000013189, ENSGALG00000028297, ENSGALG00000034628
2	87380001	87420000	40.0	0.65	0.17	ENSGALG00000030991
2	88100001	88160000	60.0	0.61	0.13	ENSGALG00000013196, ENSGALG00000013197
2	88340001	88720000	380.0	1.17	0.16	ENSGALG00000013200, ENSGALG00000039034, ENSGALG00000028986
2	107500001	107560000	60.0	0.50	0.13	ENSGALG00000015198
2	110080001	110120000	40.0	0.61	0.20	ENSGALG00000015254
2	110660001	110740000	80.0	0.52	0.21	ENSGALG00000015274, ENSGALG00000040421, ENSGALG00000025941, ENSGALG00000041086
2	113640001	113680000	40.0	0.60	0.14	ENSGALG00000030112, ENSGALG00000043172, ENSGALG00000043440
2	115160001	115240000	80.0	0.47	0.18	ENSGALG00000038385, ENSGALG00000036567, ENSGALG00000038395
2	118900001	118980000	80.0	0.68	0.15	ENSGALG00000033880, ENSGALG00000041571
2	120160001	120200000	40.0	0.59	0.13	ENSGALG00000036262
2	121720001	121760000	40.0	1.11	0.15	ENSGALG00000034188
2	139180001	139240000	60.0	0.90	0.16	ENSGALG00000032651
2	148740001	148780000	40.0	0.46	0.13	ENSGALG00000043265, ENSGALG00000026264
3	20320001	20360000	40.0	0.53	0.21	ENSGALG00000009645
3	29020001	29180000	160.0	1.16	0.22	ENSGALG00000010057, ENSGALG00000010068, ENSGALG00000020049
3	29640001	29720000	80.0	0.52	0.17	ENSGALG00000010131
3	32400001	32520000	120.0	1.03	0.18	ENSGALG00000032640
3	38220001	38280000	60.0	0.68	0.14	ENSGALG00000034003, ENSGALG00000042853
3	43820001	43860000	40.0	0.75	0.17	ENSGALG00000032211

3	50060001	50160000	100.0	2.33	0.23	ENSGALG00000036112
3	57440001	57520000	80.0	0.59	0.19	ENSGALG00000037587, ENSGALG00000040607, ENSGALG00000029961
3	57660001	57800000	140.0	0.60	0.20	ENSGALG00000035769
3	61520001	61680000	160.0	0.68	0.14	ENSGALG00000041356
3	69060001	69100000	40.0	0.78	0.13	ENSGALG00000015410, ENSGALG00000026761
3	80440001	80480000	40.0	1.41	0.14	ENSGALG00000041312
3	81060001	81100000	40.0	0.62	0.13	ENSGALG00000015908, ENSGALG00000015904, ENSGALG00000015906, ENSGALG00000029372
3	85200001	85240000	40.0	0.52	0.13	ENSGALG00000039997
3	86540001	86600000	60.0	0.48	0.16	ENSGALG00000016276
3	93760001	93800000	40.0	0.70	0.17	ENSGALG00000031268
3	94080001	94120000	40.0	0.58	0.14	ENSGALG00000016392, ENSGALG00000016389, ENSGALG00000037309, ENSGALG00000032164, ENSGALG00000025796
3	100440001	100480000	40.0	2.13	0.19	ENSGALG00000044890
3	110980001	111020000	40.0	0.81	0.13	ENSGALG00000030006, ENSGALG00000037267
4	5960001	6020000	60.0	0.85	0.15	ENSGALG00000030791
4	23960001	24080000	120.0	0.88	0.14	ENSGALG00000009554, ENSGALG00000027788, ENSGALG00000009528, ENSGALG00000017930, ENSGALG00000009560
4	25300001	25340000	40.0	0.53	0.14	ENSGALG00000009650
4	27820001	27860000	40.0	1.10	0.17	ENSGALG00000035886
4	30500001	30560000	60.0	0.83	0.14	ENSGALG00000009880
4	33980001	34020000	40.0	0.68	0.13	ENSGALG00000010103
4	40020001	40100000	80.0	0.48	0.15	ENSGALG00000010654, ENSGALG00000010658, ENSGALG00000010659, ENSGALG00000033015, ENSGALG00000010661, ENSGALG00000025557
4	42300001	42440000	140.0	0.57	0.24	ENSGALG00000043173
4	43960001	44000000	40.0	0.79	0.14	ENSGALG00000010764, ENSGALG00000020201
4	46360001	46420000	60.0	0.46	0.15	ENSGALG00000011124
4	50060001	50100000	40.0	0.60	0.15	ENSGALG00000042427

4	50220001	50260000	40.0	0.51	0.22	ENSGALG00000011499, ENSGALG00000027872
4	57080001	57240000	160.0	0.51	0.20	ENSGALG00000012044
4	60360001	60400000	40.0	0.48	0.14	ENSGALG00000030614, ENSGALG00000020160, ENSGALG00000012264
4	63340001	63420000	80.0	1.34	0.27	ENSGALG00000013615, ENSGALG00000013617
4	64940001	65000000	60.0	0.85	0.15	ENSGALG00000013715
4	65800001	65840000	40.0	0.55	0.15	ENSGALG00000013929, ENSGALG00000038607, ENSGALG00000044908
4	66180001	66220000	40.0	0.69	0.15	ENSGALG00000039168
4	66580001	66620000	40.0	0.56	0.15	ENSGALG00000014078, ENSGALG00000013964, ENSGALG00000013968
4	67060001	67220000	160.0	0.62	0.18	ENSGALG00000014144, ENSGALG00000014192
4	70640001	70740000	100.0	0.56	0.15	ENSGALG00000041373
4	71460001	71760000	300.0	1.23	0.32	ENSGALG00000030680
4	72020001	72120000	100.0	1.06	0.14	ENSGALG00000033883
4	86000001	86060000	60.0	0.56	0.14	ENSGALG00000038160, ENSGALG00000015773, ENSGALG00000015783, ENSGALG00000017860
4	86080001	86120000	40.0	0.53	0.15	ENSGALG00000015786
4	86500001	86540000	40.0	0.80	0.15	ENSGALG00000015935, ENSGALG00000015939, ENSGALG00000046009, ENSGALG00000015937
5	5100001	5140000	40.0	0.49	0.13	ENSGALG00000012127
5	13100001	13140000	40.0	0.74	0.14	ENSGALG00000006446, ENSGALG00000006427, ENSGALG00000032687, ENSGALG00000024572, ENSGALG00000025103
5	28360001	28400000	40.0	0.60	0.15	ENSGALG00000009487, ENSGALG00000009491
5	32220001	32300000	80.0	1.11	0.19	ENSGALG00000009844, ENSGALG00000009838, ENSGALG00000009845
5	32400001	32460000	60.0	1.98	0.28	ENSGALG00000009847
5	32840001	32980000	140.0	0.82	0.15	ENSGALG00000009850
5	46560001	46600000	40.0	0.60	0.17	ENSGALG00000033099
5	58480001	58540000	60.0	1.02	0.16	ENSGALG00000012426

6	8200001	8240000	40.0	0.72	0.13	ENSGALG00000002980, ENSGALG00000002967
6	9020001	9080000	60.0	0.70	0.16	ENSGALG00000003135, ENSGALG00000029580
6	9440001	9480000	40.0	0.76	0.14	ENSGALG00000029829, ENSGALG00000046392
6	14800001	14840000	40.0	0.47	0.15	ENSGALG00000005000, ENSGALG00000005002
6	14960001	15080000	120.0	0.69	0.15	ENSGALG00000005035, ENSGALG00000005019
6	17640001	17680000	40.0	0.62	0.13	ENSGALG00000005867, ENSGALG00000005857
6	18160001	18220000	60.0	0.48	0.15	ENSGALG00000006095
6	18380001	18580000	200.0	1.85	0.16	ENSGALG00000036693, ENSGALG00000006165, ENSGALG00000006174, ENSGALG00000006168
6	18980001	19040000	60.0	0.91	0.17	ENSGALG00000041346
6	19100001	19220000	120.0	0.70	0.19	ENSGALG00000026808, ENSGALG00000023824, ENSGALG00000006302, ENSGALG00000028250, ENSGALG00000006331
6	19240001	19300000	60.0	0.67	0.13	ENSGALG00000006351, ENSGALG00000006343
6	19400001	19440000	40.0	0.56	0.14	ENSGALG00000006431, ENSGALG00000034232, ENSGALG00000018330
6	19700001	19740000	40.0	0.59	0.16	ENSGALG00000006502
6	20780001	20820000	40.0	0.62	0.13	ENSGALG00000032234, ENSGALG00000038924, ENSGALG00000030061
6	21260001	21320000	60.0	0.70	0.18	ENSGALG00000006983
7	5800001	5900000	100.0	0.77	0.15	ENSGALG00000030235, ENSGALG00000026808, ENSGALG00000023824, ENSGALG00000006302, ENSGALG00000028250, ENSGALG00000006331, ENSGALG00000004196, ENSGALG00000023761, ENSGALG00000004228, ENSGALG00000043856, ENSGALG00000044780, ENSGALG00000036951,

						ENSGALG00000044892, ENSGALG00000031157
7	9640001	9680000	40.0	0.73	0.19	ENSGALG00000007777, ENSGALG00000007841, ENSGALG00000035909
7	12020001	12060000	40.0	0.51	0.16	ENSGALG00000008474, ENSGALG00000008470
7	15020001	15060000	40.0	0.81	0.18	ENSGALG00000009012
7	17000001	17040000	40.0	0.90	0.14	ENSGALG00000009325, ENSGALG00000043079, ENSGALG00000014276
7	19680001	19720000	40.0	0.58	0.13	ENSGALG00000010943
7	29420001	29520000	100.0	1.02	0.20	ENSGALG00000012156, ENSGALG00000037581
7	30500001	30560000	60.0	0.56	0.18	ENSGALG00000012190
8	8320001	8360000	40.0	0.50	0.14	ENSGALG00000033407
8	9500001	9540000	40.0	2.03	0.13	ENSGALG00000043258
8	9840001	9920000	80.0	0.88	0.20	ENSGALG00000033635, ENSGALG00000005065
8	11860001	11900000	40.0	0.51	0.14	ENSGALG00000005280, ENSGALG00000005257, ENSGALG00000005277, ENSGALG00000045129, ENSGALG00000005284
8	12300001	12420000	120.0	0.88	0.19	ENSGALG00000005470, ENSGALG00000005478
8	12520001	12560000	40.0	0.74	0.14	ENSGALG00000034994, ENSGALG00000005509
8	12620001	12680000	60.0	0.52	0.21	ENSGALG00000005509
8	12900001	12940000	40.0	0.93	0.13	ENSGALG00000005552
8	19080001	19120000	40.0	0.92	0.15	ENSGALG00000008988
8	24820001	24860000	40.0	1.11	0.16	ENSGALG00000010692
8	26720001	26780000	60.0	0.80	0.18	ENSGALG00000010889, ENSGALG00000011394, ENSGALG00000045609
9	8740001	8780000	40.0	0.47	0.14	ENSGALG00000004966, ENSGALG00000031808
9	11480001	11560000	80.0	2.16	0.30	ENSGALG00000006783
9	17620001	17660000	40.0	0.55	0.18	ENSGALG00000041873, ENSGALG00000040948, ENSGALG00000031929

9	23480001	23520000	40.0	1.03	0.15	ENSGALG00000029190
10	6320001	6480000	160.0	0.79	0.18	ENSGALG00000004087
10	7840001	7880000	40.0	0.88	0.15	ENSGALG00000037336
11	2340001	2380000	40.0	0.51	0.14	ENSGALG00000003163, ENSGALG00000035175, ENSGALG00000036972
11	2520001	2600000	80.0	0.69	0.17	ENSGALG00000003224, ENSGALG00000003293, ENSGALG00000003258, ENSGALG00000003309, ENSGALG00000003273, ENSGALG00000003302, ENSGALG00000003201
11	6000001	6040000	40.0	0.93	0.17	ENSGALG00000039238
11	6800001	6860000	60.0	0.76	0.14	ENSGALG00000003891, ENSGALG00000045466
11	8480001	8560000	80.0	0.47	0.13	ENSGALG00000029853, ENSGALG00000004489, ENSGALG00000004480, ENSGALG00000032626, ENSGALG00000032428, ENSGALG00000030532, ENSGALG00000041972, ENSGALG00000004474
11	8880001	8920000	40.0	0.59	0.14	ENSGALG00000035602
11	10520001	10560000	40.0	0.70	0.15	ENSGALG00000004899
11	13980001	14020000	40.0	0.48	0.13	ENSGALG00000005347
12	900001	940000	40.0	0.47	0.16	ENSGALG00000001753
12	10620001	10660000	40.0	0.48	0.14	ENSGALG00000036133, ENSGALG00000042231, ENSGALG00000044610, ENSGALG00000034962
12	14000001	14040000	40.0	0.53	0.14	ENSGALG00000007431
13	1220001	1300000	80.0	0.49	0.17	ENSGALG00000033641, ENSGALG00000030836
13	4100001	4140000	40.0	0.78	0.13	ENSGALG00000002080, ENSGALG00000001942
14	8860001	8940000	80.0	1.43	0.16	ENSGALG00000007130, ENSGALG00000007149, ENSGALG00000007133, ENSGALG00000042905
14	14760001	14820000	60.0	0.86	0.25	ENSGALG00000002119, ENSGALG00000002134, ENSGALG00000002200
15	3500001	3640000	140.0	0.67	0.24	ENSGALG00000002671

15	6200001	6240000	40.0	0.61	0.14	ENSGALG00000004625, ENSGALG00000004650
17	4560001	4600000	40.0	0.59	0.17	ENSGALG00000006997, ENSGALG00000039255
18	3480001	3520000	40.0	0.73	0.13	ENSGALG00000001577, ENSGALG00000001603, ENSGALG00000034018, ENSGALG00000017910
19	2480001	2520000	40.0	0.48	0.13	ENSGALG00000036742
19	9880001	9920000	40.0	0.70	0.14	ENSGALG00000006034, ENSGALG00000006064, ENSGALG00000029322, ENSGALG00000006048
20	320001	360000	40.0	0.52	0.14	ENSGALG00000001046
20	3120001	3180000	60.0	0.52	0.18	ENSGALG00000037889, ENSGALG00000037613
20	6440001	6500000	60.0	0.54	0.14	ENSGALG00000004621, ENSGALG00000029984
20	6540001	6620000	80.0	0.70	0.17	ENSGALG00000004663, ENSGALG00000004707, ENSGALG00000004736
20	10860001	10920000	60.0	0.67	0.17	ENSGALG00000007211, ENSGALG00000020939
20	13440001	13480000	40.0	0.71	0.13	ENSGALG00000007963
21	3560001	3600000	40.0	1.15	0.13	ENSGALG00000002649, ENSGALG00000002616, ENSGALG00000043318, ENSGALG00000002637
21	3680001	3720000	40.0	0.88	0.15	ENSGALG00000002726, ENSGALG00000002797, ENSGALG00000024481
21	4600001	4640000	40.0	0.64	0.13	ENSGALG00000043204, ENSGALG00000030563, ENSGALG00000029132
21	4660001	4720000	60.0	0.62	0.14	ENSGALG00000004034, ENSGALG00000003969, ENSGALG00000033204, ENSGALG00000004005, ENSGALG00000003991
22	840001	880000	40.0	0.57	0.23	ENSGALG00000000311
24	6080001	6160000	80.0	1.20	0.25	ENSGALG00000007868, ENSGALG00000043837, ENSGALG00000007878, ENSGALG00000007885, ENSGALG00000007848,

						ENSGALG00000007874, ENSGALG00000007904, ENSGALG00000007882, ENSGALG00000007929
25	1040001	1100000	60.0	0.50	0.19	ENSGALG00000024138, ENSGALG00000009153, ENSGALG00000045026, ENSGALG00000029144, ENSGALG00000024136, ENSGALG00000018882, ENSGALG00000030867, ENSGALG00000042507, ENSGALG00000035665, ENSGALG00000032276, ENSGALG00000042032, ENSGALG00000043207, ENSGALG00000030486, ENSGALG00000038929, ENSGALG00000031300, ENSGALG00000033482
26	1320001	1380000	60.0	0.80	0.19	ENSGALG00000000400, ENSGALG00000000394, ENSGALG00000046044, ENSGALG00000037995, ENSGALG00000000401
26	1480001	1540000	60.0	0.69	0.16	ENSGALG00000000483, ENSGALG00000000511, ENSGALG00000000477, ENSGALG00000036942, ENSGALG00000000504, ENSGALG00000000497

Supplementary Table S8 Selected candidate genes by using log2 θπ ratio and F_{ST}

Gene ID
ENSGALG00000000311
ENSGALG00000000394
ENSGALG00000000400
ENSGALG00000000401
ENSGALG00000000477
ENSGALG00000000483
ENSGALG00000000497
ENSGALG00000000504
ENSGALG00000000511
ENSGALG00000001046
ENSGALG00000001577
ENSGALG00000001603
ENSGALG00000001753
ENSGALG00000001942
ENSGALG00000002080
ENSGALG00000002119
ENSGALG00000002134
ENSGALG00000002200
ENSGALG00000002616
ENSGALG00000002637
ENSGALG00000002649
ENSGALG00000002671
ENSGALG00000002726
ENSGALG00000002797
ENSGALG00000002967
ENSGALG00000002980
ENSGALG00000003135
ENSGALG00000003163
ENSGALG00000003201
ENSGALG00000003224
ENSGALG00000003258
ENSGALG00000003273
ENSGALG00000003293
ENSGALG00000003302
ENSGALG00000003309
ENSGALG00000003891
ENSGALG00000003969
ENSGALG00000003991
ENSGALG00000004005
ENSGALG00000004034
ENSGALG00000004087
ENSGALG00000004196

ENSGALG00000004228
ENSGALG00000004474
ENSGALG00000004480
ENSGALG00000004489
ENSGALG00000004621
ENSGALG00000004625
ENSGALG00000004650
ENSGALG00000004663
ENSGALG00000004707
ENSGALG00000004736
ENSGALG00000004899
ENSGALG00000004966
ENSGALG00000005000
ENSGALG00000005002
ENSGALG00000005019
ENSGALG00000005035
ENSGALG00000005065
ENSGALG00000005257
ENSGALG00000005277
ENSGALG00000005280
ENSGALG00000005284
ENSGALG00000005347
ENSGALG00000005470
ENSGALG00000005478
ENSGALG00000005509
ENSGALG00000005552
ENSGALG00000005857
ENSGALG00000005867
ENSGALG00000006034
ENSGALG00000006048
ENSGALG00000006064
ENSGALG00000006095
ENSGALG00000006165
ENSGALG00000006168
ENSGALG00000006174
ENSGALG00000006302
ENSGALG00000006331
ENSGALG00000006343
ENSGALG00000006351
ENSGALG00000006427
ENSGALG00000006431
ENSGALG00000006446
ENSGALG00000006502
ENSGALG00000006783
ENSGALG00000006983

ENSGALG00000006997
ENSGALG00000007125
ENSGALG00000007130
ENSGALG00000007133
ENSGALG00000007140
ENSGALG00000007149
ENSGALG00000007211
ENSGALG00000007431
ENSGALG00000007777
ENSGALG00000007841
ENSGALG00000007848
ENSGALG00000007868
ENSGALG00000007874
ENSGALG00000007878
ENSGALG00000007882
ENSGALG00000007885
ENSGALG00000007904
ENSGALG00000007929
ENSGALG00000007963
ENSGALG00000008470
ENSGALG00000008474
ENSGALG00000008486
ENSGALG00000008497
ENSGALG00000008988
ENSGALG00000009012
ENSGALG00000009153
ENSGALG00000009325
ENSGALG00000009487
ENSGALG00000009491
ENSGALG00000009528
ENSGALG00000009554
ENSGALG00000009560
ENSGALG00000009645
ENSGALG00000009650
ENSGALG00000009698
ENSGALG00000009838
ENSGALG00000009844
ENSGALG00000009845
ENSGALG00000009847
ENSGALG00000009850
ENSGALG00000009880
ENSGALG00000010057
ENSGALG00000010068
ENSGALG00000010103
ENSGALG00000010131

ENSGALG00000010654
ENSGALG00000010658
ENSGALG00000010659
ENSGALG00000010661
ENSGALG00000010692
ENSGALG00000010718
ENSGALG00000010764
ENSGALG00000010836
ENSGALG00000010840
ENSGALG00000010889
ENSGALG00000010935
ENSGALG00000010943
ENSGALG00000011124
ENSGALG00000011269
ENSGALG00000011270
ENSGALG00000011271
ENSGALG00000011285
ENSGALG00000011295
ENSGALG00000011297
ENSGALG00000011394
ENSGALG00000011499
ENSGALG00000011854
ENSGALG00000011930
ENSGALG00000012044
ENSGALG00000012127
ENSGALG00000012156
ENSGALG00000012190
ENSGALG00000012264
ENSGALG00000012426
ENSGALG00000012715
ENSGALG00000012739
ENSGALG00000012755
ENSGALG00000012760
ENSGALG00000012761
ENSGALG00000012791
ENSGALG00000012941
ENSGALG00000012964
ENSGALG00000013102
ENSGALG00000013180
ENSGALG00000013183
ENSGALG00000013187
ENSGALG00000013189
ENSGALG00000013196
ENSGALG00000013197
ENSGALG00000013200

ENSGALG00000013615
ENSGALG00000013617
ENSGALG00000013715
ENSGALG00000013929
ENSGALG00000013964
ENSGALG00000013968
ENSGALG00000014078
ENSGALG00000014144
ENSGALG00000014178
ENSGALG00000014192
ENSGALG00000014276
ENSGALG00000014329
ENSGALG00000015198
ENSGALG00000015254
ENSGALG00000015267
ENSGALG00000015274
ENSGALG00000015410
ENSGALG00000015770
ENSGALG00000015773
ENSGALG00000015783
ENSGALG00000015786
ENSGALG00000015904
ENSGALG00000015906
ENSGALG00000015908
ENSGALG00000015935
ENSGALG00000015937
ENSGALG00000015939
ENSGALG00000016044
ENSGALG00000016054
ENSGALG00000016276
ENSGALG00000016382
ENSGALG00000016388
ENSGALG00000016389
ENSGALG00000016391
ENSGALG00000016392
ENSGALG00000016828
ENSGALG00000016860
ENSGALG00000016861
ENSGALG00000016862
ENSGALG00000016863
ENSGALG00000016868
ENSGALG00000016902
ENSGALG00000017111
ENSGALG00000017112
ENSGALG00000017228

ENSGALG00000017271
ENSGALG00000017272
ENSGALG00000017280
ENSGALG00000017282
ENSGALG00000017283
ENSGALG00000017860
ENSGALG00000017910
ENSGALG00000017930
ENSGALG00000018330
ENSGALG00000018882
ENSGALG00000019157
ENSGALG00000019361
ENSGALG00000019527
ENSGALG00000020049
ENSGALG00000020160
ENSGALG00000020201
ENSGALG00000020939
ENSGALG00000023584
ENSGALG00000023761
ENSGALG00000023824
ENSGALG00000024136
ENSGALG00000024138
ENSGALG00000024481
ENSGALG00000024572
ENSGALG00000025103
ENSGALG00000025557
ENSGALG00000025619
ENSGALG00000025796
ENSGALG00000025941
ENSGALG00000026264
ENSGALG00000026372
ENSGALG00000026409
ENSGALG00000026661
ENSGALG00000026761
ENSGALG00000026808
ENSGALG00000026901
ENSGALG00000027620
ENSGALG00000027788
ENSGALG00000027816
ENSGALG00000027872
ENSGALG00000027893
ENSGALG00000028250
ENSGALG00000028297
ENSGALG00000028986
ENSGALG00000029132

ENSGALG00000029144
ENSGALG00000029190
ENSGALG00000029322
ENSGALG00000029372
ENSGALG00000029580
ENSGALG00000029785
ENSGALG00000029829
ENSGALG00000029853
ENSGALG00000029961
ENSGALG00000029984
ENSGALG00000030006
ENSGALG00000030061
ENSGALG00000030112
ENSGALG00000030235
ENSGALG00000030486
ENSGALG00000030532
ENSGALG00000030557
ENSGALG00000030563
ENSGALG00000030614
ENSGALG00000030680
ENSGALG00000030791
ENSGALG00000030836
ENSGALG00000030867
ENSGALG00000030991
ENSGALG00000031157
ENSGALG00000031268
ENSGALG00000031294
ENSGALG00000031300
ENSGALG00000031323
ENSGALG00000031503
ENSGALG00000031713
ENSGALG00000031751
ENSGALG00000031758
ENSGALG00000031808
ENSGALG00000031929
ENSGALG00000032164
ENSGALG00000032211
ENSGALG00000032234
ENSGALG00000032276
ENSGALG00000032339
ENSGALG00000032428
ENSGALG00000032626
ENSGALG00000032640
ENSGALG00000032651
ENSGALG00000032683

ENSGALG00000032687
ENSGALG00000033015
ENSGALG00000033099
ENSGALG00000033204
ENSGALG00000033407
ENSGALG00000033482
ENSGALG00000033533
ENSGALG00000033635
ENSGALG00000033641
ENSGALG00000033880
ENSGALG00000033883
ENSGALG00000033942
ENSGALG00000034003
ENSGALG00000034018
ENSGALG00000034188
ENSGALG00000034232
ENSGALG00000034312
ENSGALG00000034616
ENSGALG00000034628
ENSGALG00000034962
ENSGALG00000034994
ENSGALG00000035126
ENSGALG00000035175
ENSGALG00000035602
ENSGALG00000035665
ENSGALG00000035769
ENSGALG00000035886
ENSGALG00000035909
ENSGALG00000035959
ENSGALG00000036110
ENSGALG00000036112
ENSGALG00000036133
ENSGALG00000036149
ENSGALG00000036190
ENSGALG00000036222
ENSGALG00000036262
ENSGALG00000036567
ENSGALG00000036693
ENSGALG00000036730
ENSGALG00000036742
ENSGALG00000036942
ENSGALG00000036951
ENSGALG00000036972
ENSGALG00000037267
ENSGALG00000037309

ENSGALG00000037336
ENSGALG00000037581
ENSGALG00000037587
ENSGALG00000037613
ENSGALG00000037656
ENSGALG00000037697
ENSGALG00000037889
ENSGALG00000037995
ENSGALG00000038160
ENSGALG00000038385
ENSGALG00000038395
ENSGALG00000038439
ENSGALG00000038607
ENSGALG00000038924
ENSGALG00000038929
ENSGALG00000039004
ENSGALG00000039034
ENSGALG00000039168
ENSGALG00000039238
ENSGALG00000039255
ENSGALG00000039442
ENSGALG00000039534
ENSGALG00000039935
ENSGALG00000039997
ENSGALG00000040266
ENSGALG00000040421
ENSGALG00000040444
ENSGALG00000040607
ENSGALG00000040645
ENSGALG00000040681
ENSGALG00000040891
ENSGALG00000040898
ENSGALG00000040948
ENSGALG00000041086
ENSGALG00000041094
ENSGALG00000041312
ENSGALG00000041346
ENSGALG00000041356
ENSGALG00000041373
ENSGALG00000041571
ENSGALG00000041873
ENSGALG00000041941
ENSGALG00000041972
ENSGALG00000042032
ENSGALG00000042231

ENSGALG00000042427
ENSGALG00000042507
ENSGALG00000042678
ENSGALG00000042836
ENSGALG00000042853
ENSGALG00000042905
ENSGALG00000043079
ENSGALG00000043172
ENSGALG00000043173
ENSGALG00000043204
ENSGALG00000043207
ENSGALG00000043258
ENSGALG00000043265
ENSGALG00000043318
ENSGALG00000043440
ENSGALG00000043800
ENSGALG00000043837
ENSGALG00000043856
ENSGALG00000044610
ENSGALG00000044780
ENSGALG00000044890
ENSGALG00000044892
ENSGALG00000044908
ENSGALG00000045026
ENSGALG00000045129
ENSGALG00000045240
ENSGALG00000045466
ENSGALG00000045609
ENSGALG00000045907
ENSGALG00000045908
ENSGALG00000046009
ENSGALG00000046044
ENSGALG00000046372
ENSGALG00000046392

Supplementary Table S9 Genomic regions with strong selective sweep signals in NYs defined by XP-CLR (top 1% outliers)

Chromosome	Location Start (bp)	Location End (bp)	Selective Sweep Length (kb)	XP-CLR score	Gene ID
1	385001	405000	20.0	31.637	ENSGALG00000039165
1	735001	750000	15.0	27.613	ENSGALG00000032868
1	11200001	11215000	15.0	29.959	ENSGALG00000008439
1	18780001	18795000	15.0	24.554	ENSGALG00000029379
1	30425001	30440000	15.0	32.555	ENSGALG00000009601
1	31015001	31030000	15.0	81.981	ENSGALG00000025619
1	31040001	31050000	10.0	104.99	ENSGALG00000043800
1	31105001	31120000	15.0	39.81	ENSGALG00000009730
1	34065001	34075000	10.0	27.738	ENSGALG00000026661
1	38195001	38210000	15.0	24.34	ENSGALG00000010253
1	38660001	38670000	10.0	26.899	ENSGALG00000010311
1	39285001	39300000	15.0	26.77	ENSGALG00000041094
1	39865001	39880000	15.0	71.011	ENSGALG00000010935
1	43945001	43955000	10.0	32.626	ENSGALG00000011269
1	43995001	44005000	10.0	67.606	ENSGALG00000011271
1	44035001	44065000	30.0	24.865	ENSGALG00000011274
1	44905001	44920000	15.0	88.957	ENSGALG00000011297
1	49090001	49125000	35.0	51.841	ENSGALG00000011854
1	50040001	50055000	15.0	25.317	ENSGALG00000012024
1	52820001	52835000	15.0	65.176	ENSGALG00000012559
1	55195001	55210000	15.0	25.947	ENSGALG00000019300
1	55895001	55910000	15.0	36.479	ENSGALG00000012791
1	56175001	56190000	15.0	57.18	ENSGALG00000012792
1	65190001	65205000	15.0	42.64	ENSGALG00000013154
1	68770001	68795000	25.0	61.302	ENSGALG00000014178
1	71860001	71880000	20.0	25.164	ENSGALG00000031503
1	73280001	73290000	10.0	44.671	ENSGALG00000017283
1	73425001	73495000	70.0	22.872	ENSGALG00000033533
1	76380001	76395000	15.0	38.329	ENSGALG00000011930
1	77270001	77285000	15.0	50.988	ENSGALG00000014525, ENSGALG00000014526, ENSGALG00000043468
1	84555001	84570000	15.0	35.571	ENSGALG00000015258
1	84605001	84620000	15.0	76.328	ENSGALG00000015267
1	88555001	88570000	15.0	55.09	ENSGALG00000015378, ENSGALG00000032712
1	88615001	88630000	15.0	28.744	ENSGALG00000015389
1	88635001	88660000	25.0	37.596	ENSGALG00000019221

1	88685001	88700000	15.0	55.333	ENSGALG00000045596
1	95645001	95655000	10.0	33.449	ENSGALG00000025630
1	102680001	102695000	15.0	23.123	ENSGALG00000015770
1	106795001	106805000	10.0	89.18	ENSGALG00000016044
1	106860001	106885000	25.0	63.41	ENSGALG00000016047
1	112130001	112145000	15.0	30.952	ENSGALG00000016236
1	115280001	115295000	15.0	25.23	ENSGALG00000016281
1	115910001	115920000	10.0	36.953	ENSGALG00000016287
1	119060001	119075000	15.0	47.246	ENSGALG00000016382
1	119140001	119155000	15.0	27.894	ENSGALG00000019157
1	121450001	121465000	15.0	27.232	ENSGALG00000016548, ENSGALG00000016549
1	129080001	129095000	15.0	44.526	ENSGALG00000016686
1	132540001	132555000	15.0	23.439	ENSGALG00000016758, ENSGALG00000016757, ENSGALG00000016759
1	134860001	134885000	25.0	30.334	ENSGALG00000042484
1	137120001	137135000	15.0	37.143	ENSGALG00000016817
1	137460001	137505000	45.0	124.312	ENSGALG00000016828, ENSGALG00000037697
1	142780001	142795000	15.0	54.054	ENSGALG00000016861, ENSGALG00000016862
1	143650001	143695000	45.0	121.822	ENSGALG00000016868
1	147825001	147920000	95.0	61.237	ENSGALG00000016902
1	148415001	148430000	15.0	25.826	ENSGALG00000038439
1	148545001	148565000	20.0	40.45	ENSGALG00000045327
1	160705001	160890000	185.0	30.189	ENSGALG00000045907, ENSGALG00000040645
1	161755001	161770000	15.0	37.359	ENSGALG00000030557, ENSGALG00000040645
1	167965001	167980000	15.0	25.233	ENSGALG00000016984
1	174265001	174280000	15.0	23.959	ENSGALG00000017068
1	178430001	178440000	10.0	42.969	ENSGALG00000017125
1	184405001	184420000	15.0	27.822	ENSGALG00000042076
2	315001	330000	15.0	26.598	ENSGALG00000042836
2	4525001	4540000	15.0	26.128	ENSGALG00000005710
2	5655001	5665000	10.0	28.005	ENSGALG00000006112
2	11430001	11455000	25.0	40.869	ENSGALG00000006939
2	13220001	13235000	15.0	58.826	ENSGALG00000007125
2	13890001	13975000	85.0	70.24	ENSGALG00000007140
2	15670001	15680000	10.0	47.135	ENSGALG00000007417
2	16860001	16875000	15.0	29.581	ENSGALG00000007721
2	16970001	16985000	15.0	32.165	ENSGALG00000007766
2	20090001	20105000	15.0	69.543	ENSGALG00000040891
2	20140001	20155000	15.0	24.573	ENSGALG00000032554

2	24525001	24540000	15.0	44.123	ENSGALG00000027620
2	26375001	26430000	55.0	26.291	ENSGALG00000010718
2	28835001	28850000	15.0	58.789	ENSGALG00000010836
2	29045001	29080000	35.0	23.462	ENSGALG00000010840
2	38010001	38025000	15.0	36.51	ENSGALG00000011300
2	43645001	43660000	15.0	32.708	ENSGALG00000036110
2	43875001	43890000	15.0	44.844	ENSGALG00000038733
2	45565001	45580000	15.0	41.945	ENSGALG00000025546
2	48515001	48530000	15.0	79.01	ENSGALG00000039935
2	49705001	49870000	165.0	37.192	ENSGALG00000031713, ENSGALG00000034312
2	50155001	50200000	45.0	27.614	ENSGALG00000031758
2	50740001	50750000	10.0	23.199	ENSGALG00000034616
2	61510001	61525000	15.0	37.984	ENSGALG00000012703
2	62915001	62930000	15.0	24.872	ENSGALG00000012739
2	67625001	67640000	15.0	26.537	ENSGALG00000033170
2	70855001	70870000	15.0	46.907	ENSGALG00000031547
2	76385001	76450000	65.0	31.329	ENSGALG00000012964
2	78055001	78070000	15.0	25.297	ENSGALG00000013001, ENSGALG00000025806
2	78495001	78510000	15.0	24.698	ENSGALG00000013006, ENSGALG00000013005, ENSGALG00000035699
2	79965001	79980000	15.0	27.474	ENSGALG00000013061
2	81405001	81485000	80.0	58.609	ENSGALG00000013102
2	83365001	83380000	15.0	24.544	ENSGALG00000013124
2	85065001	85080000	15.0	38.796	ENSGALG00000013149
2	85965001	85990000	25.0	99.311	ENSGALG00000019527
2	86045001	86055000	10.0	28.669	ENSGALG00000013183
2	86075001	86090000	15.0	54.217	ENSGALG00000013187, ENSGALG00000028297
2	86740001	86750000	10.0	31.532	ENSGALG00000025484
2	86895001	86915000	20.0	52.067	ENSGALG00000013193
2	87400001	87410000	10.0	34.956	ENSGALG00000030991
2	88135001	88150000	15.0	23.205	ENSGALG00000013196
2	88445001	88530000	85.0	26.075	ENSGALG00000013200
2	92245001	92260000	15.0	27.788	ENSGALG00000013708
2	98290001	98305000	15.0	24.058	ENSGALG00000013932, ENSGALG00000022875
2	106705001	106730000	25.0	79.696	ENSGALG00000031453, ENSGALG00000044606
2	107515001	107530000	15.0	65.382	ENSGALG00000015198
2	110675001	110700000	25.0	121.845	ENSGALG00000015274, ENSGALG00000025941
2	113565001	113580000	15.0	28.394	ENSGALG00000043172

2	113645001	113660000	15.0	24.737	ENSGALG00000030112, ENSGALG00000043440
2	115185001	115240000	55.0	46.515	ENSGALG00000036567, ENSGALG00000038385, ENSGALG00000038395
2	118915001	118955000	40.0	74.891	ENSGALG00000033880
2	118980001	118990000	10.0	55.189	ENSGALG00000041571
2	121735001	121770000	35.0	46.102	ENSGALG00000034188
2	126875001	126890000	15.0	23.6	ENSGALG00000033089
2	128315001	128330000	15.0	46.426	ENSGALG00000032850
2	129235001	129250000	15.0	30.466	ENSGALG00000031387
2	132215001	132230000	15.0	26.382	ENSGALG00000030030
2	138945001	138960000	15.0	26.585	ENSGALG00000043439
2	139210001	139220000	10.0	24.115	ENSGALG00000032651
2	141495001	141510000	15.0	38.789	ENSGALG00000039224
2	141575001	141610000	35.0	23.614	ENSGALG00000037626
2	145275001	145285000	10.0	45.521	ENSGALG00000032103
2	147215001	147360000	145.0	26.244	ENSGALG00000037014
2	149525001	149540000	15.0	26.135	ENSGALG00000034392, ENSGALG00000043420, ENSGALG00000036465
3	13930001	13945000	15.0	35.4	ENSGALG00000008947, ENSGALG00000008918
3	20040001	20355000	315.0	120.098	ENSGALG00000009645
3	27055001	27070000	15.0	53.928	ENSGALG00000010020
3	29015001	29140000	125.0	79.107	ENSGALG00000010057
3	29395001	29410000	15.0	24.492	ENSGALG00000010078
3	29625001	29640000	15.0	41.204	ENSGALG00000010131
3	30285001	30300000	15.0	94.764	ENSGALG00000035337
3	32415001	32500000	85.0	110.125	ENSGALG00000032640
3	35150001	35195000	45.0	62.075	ENSGALG00000034081
3	38250001	38265000	15.0	109.5	ENSGALG00000034003
3	39760001	39775000	15.0	24.797	ENSGALG00000039349
3	45200001	45215000	15.0	27.183	ENSGALG00000011562
3	48245001	48255000	10.0	29.999	ENSGALG00000038782
3	48500001	48515000	15.0	98.798	ENSGALG00000041575
3	51440001	51625000	185.0	35.208	ENSGALG00000013683
3	55085001	55100000	15.0	27.477	ENSGALG00000030545, ENSGALG00000013874
3	57485001	57500000	15.0	69.597	ENSGALG00000040607
3	57580001	57650000	70.0	70.492	ENSGALG00000037109, ENSGALG00000029794, ENSGALG00000038188
3	57715001	57790000	75.0	111.508	ENSGALG00000035769
3	59055001	59070000	15.0	24.811	ENSGALG00000037423

3	61525001	61540000	15.0	28.885	ENSGALG00000041356
3	67515001	67530000	15.0	44.846	ENSGALG00000015297, ENSGALG00000039898
3	67605001	67620000	15.0	27.537	ENSGALG00000015303, ENSGALG00000027236
3	69055001	69080000	25.0	27.335	ENSGALG00000015410
3	75735001	75750000	15.0	24.604	ENSGALG00000015733
3	80425001	80460000	35.0	43.684	ENSGALG00000041312
3	83610001	83625000	15.0	23.168	ENSGALG00000030872
3	87550001	87565000	15.0	26.976	ENSGALG00000016292
3	88660001	88675000	15.0	31.501	ENSGALG00000016316, ENSGALG00000016320
3	92510001	92520000	10.0	24.229	ENSGALG00000028744
3	93745001	93755000	10.0	25.29	ENSGALG00000037681
3	94090001	94105000	15.0	38.967	ENSGALG00000016392
3	97290001	97305000	15.0	35.784	ENSGALG00000016445
3	98090001	98100000	10.0	35.886	ENSGALG00000043524
3	100030001	100045000	15.0	23.907	ENSGALG00000016463
3	107610001	107620000	10.0	23.1	ENSGALG00000016649
3	109700001	109715000	15.0	77.427	ENSGALG00000016705
4	5195001	5210000	15.0	31.367	ENSGALG00000006760, ENSGALG00000006773, ENSGALG00000006786
4	13425001	13440000	15.0	29.963	ENSGALG00000008006
4	30515001	30550000	35.0	65.24	ENSGALG00000009880
4	33245001	33260000	15.0	32.02	ENSGALG00000010061
4	33985001	34000000	15.0	44.732	ENSGALG00000010103
4	37205001	37230000	25.0	22.857	ENSGALG00000010397
4	40050001	40070000	20.0	116.684	ENSGALG00000010654, ENSGALG00000010658
4	42320001	42420000	100.0	56.664	ENSGALG00000043173
4	42450001	42460000	10.0	73.609	ENSGALG00000036597
4	42530001	42545000	15.0	45.994	ENSGALG00000010719
4	43470001	43485000	15.0	26.932	ENSGALG00000030588
4	43970001	43985000	15.0	35.37	ENSGALG00000010764
4	57145001	57360000	215.0	73.748	ENSGALG00000012044
4	63370001	63410000	40.0	79.713	ENSGALG00000013615
4	64105001	64120000	15.0	28.123	ENSGALG00000013674
4	64965001	64980000	15.0	53.99	ENSGALG00000013715
4	65805001	65820000	15.0	25.062	ENSGALG00000013929
4	67115001	67190000	75.0	120.488	ENSGALG00000014144
4	67205001	67220000	15.0	23.792	ENSGALG00000014192
4	68015001	68030000	15.0	36.037	ENSGALG00000014210, ENSGALG00000014217
4	70685001	70730000	45.0	54.763	ENSGALG00000041373

4	71000001	71010000	10.0	36.399	ENSGALG00000044225
4	71485001	71730000	245.0	40.424	ENSGALG00000030680
4	76230001	76245000	15.0	26.627	ENSGALG00000041701
4	76440001	76455000	15.0	25.861	ENSGALG00000014421, ENSGALG00000014425
4	77900001	77915000	15.0	26.258	ENSGALG00000040945
4	86020001	86035000	15.0	33.606	ENSGALG00000038160
4	86110001	86125000	15.0	50.831	ENSGALG00000015786
4	86190001	86205000	15.0	30.079	ENSGALG00000015809, ENSGALG00000015805
4	86520001	86535000	15.0	42.678	ENSGALG00000015935, ENSGALG00000015937
4	88320001	88335000	15.0	49.879	ENSGALG00000015966
5	4690001	4705000	15.0	25.931	ENSGALG00000042795
5	5100001	5115000	15.0	39.844	ENSGALG00000012127
5	5450001	5465000	15.0	49.652	ENSGALG00000012115
5	11475001	11490000	15.0	23.12	ENSGALG00000040491
5	13115001	13130000	15.0	58.014	ENSGALG00000006446, ENSGALG00000024572, ENSGALG00000025103
5	14045001	14060000	15.0	28.903	ENSGALG00000006583
5	18780001	18795000	15.0	23.331	ENSGALG00000007817
5	25210001	25225000	15.0	25.015	ENSGALG00000008940
5	28565001	28580000	15.0	26.277	ENSGALG00000009491
5	32235001	32270000	35.0	92.862	ENSGALG00000009838
5	32305001	32315000	10.0	76.776	ENSGALG00000009845
5	32945001	32960000	15.0	31.1	ENSGALG00000009850
5	34985001	35000000	15.0	26.469	ENSGALG00000009995
5	46595001	46605000	10.0	28.517	ENSGALG00000033099
5	58480001	58495000	15.0	26.078	ENSGALG00000012426
6	6510001	6525000	15.0	37.619	ENSGALG00000002744
6	11575001	11590000	15.0	42.382	ENSGALG00000045557
6	14965001	14980000	15.0	29.861	ENSGALG00000005019
6	15035001	15050000	15.0	30.96	ENSGALG00000005035
6	18195001	18210000	15.0	55.862	ENSGALG00000006095
6	18385001	18400000	15.0	30.81	ENSGALG00000036693
6	18455001	18490000	35.0	38.501	ENSGALG00000006165
6	19185001	19200000	15.0	33.04	ENSGALG00000028250, ENSGALG00000006302
6	19265001	19275000	10.0	47.553	ENSGALG00000006351
6	19390001	19410000	20.0	72.939	ENSGALG00000006431, ENSGALG00000018330
6	20785001	20800000	15.0	29.255	ENSGALG00000038924
6	20990001	21000000	10.0	24.661	ENSGALG00000020775
6	21105001	21120000	15.0	25.087	ENSGALG00000006973

6	21275001	21290000	15.0	36.572	ENSGALG00000006983
6	22765001	22790000	25.0	42.393	ENSGALG00000043428
6	23765001	23780000	15.0	38.436	ENSGALG00000008265, ENSGALG00000008281
6	26630001	26640000	10.0	24.479	ENSGALG00000008795
6	33070001	33085000	15.0	32.259	ENSGALG00000042259
6	33365001	33375000	10.0	33.496	ENSGALG00000009823
7	2280001	2295000	15.0	30.134	ENSGALG00000002750
7	4760001	4775000	15.0	26.789	ENSGALG00000003899
7	5880001	6215000	335.0	93.205	ENSGALG00000004228
7	9640001	9685000	45.0	55.843	ENSGALG00000007777, ENSGALG00000035909, ENSGALG00000007841
7	9840001	9915000	75.0	42.186	ENSGALG00000007944
7	12040001	12055000	15.0	73.424	ENSGALG00000008474
7	17000001	17015000	15.0	45.427	ENSGALG00000031751, ENSGALG00000043079
7	17030001	17045000	15.0	25.088	ENSGALG00000009325
7	26245001	26260000	15.0	35.557	ENSGALG00000011645
7	29045001	29070000	25.0	32.152	ENSGALG00000012129
7	29435001	29480000	45.0	54.198	ENSGALG00000012156
7	29535001	29545000	10.0	29.178	ENSGALG00000037581
7	30515001	30540000	25.0	54.103	ENSGALG00000012190
8	1105001	1130000	25.0	36.581	ENSGALG00000039327
8	1315001	1330000	15.0	23.237	ENSGALG00000002090
8	6145001	6160000	15.0	56.093	ENSGALG00000004163, ENSGALG00000033634, ENSGALG00000004173
8	6215001	6230000	15.0	32.477	ENSGALG00000030357
8	18510001	18525000	15.0	48.443	ENSGALG00000008890
8	18840001	18855000	15.0	23.897	ENSGALG00000038062
8	24295001	24310000	15.0	38.712	ENSGALG00000010567
8	24825001	24850000	25.0	129.092	ENSGALG00000010692
8	26745001	26760000	15.0	47.271	ENSGALG00000010889, ENSGALG00000010889
9	3535001	3550000	15.0	29.049	ENSGALG00000006680, ENSGALG00000041631
9	6585001	6600000	15.0	26.774	ENSGALG00000032053
9	7895001	7910000	15.0	33.813	ENSGALG00000030944
9	8750001	8850000	100.0	30.852	ENSGALG00000004966
9	11500001	11545000	45.0	41.229	ENSGALG00000006783
9	16430001	16445000	15.0	50.701	ENSGALG00000008759, ENSGALG00000008785
9	17630001	17645000	15.0	23.959	ENSGALG00000040948
9	20360001	20370000	10.0	68.211	ENSGALG00000032577

9	20460001	20475000	15.0	37.728	ENSGALG00000009458
9	23480001	23505000	25.0	27.743	ENSGALG00000029190
10	6380001	6425000	45.0	37.34	ENSGALG00000004087
10	6770001	6785000	15.0	25.373	ENSGALG00000004171
10	7860001	7875000	15.0	31.229	ENSGALG00000037336
10	12835001	12850000	15.0	29.462	ENSGALG00000006631, ENSGALG00000006611
10	17125001	17160000	35.0	25.945	ENSGALG00000038688
11	1760001	1775000	15.0	30.677	ENSGALG00000002467, ENSGALG00000045042
11	6035001	6045000	10.0	24.842	ENSGALG00000039238
11	6820001	6845000	25.0	66.22	ENSGALG00000003891
11	8510001	8525000	15.0	51.176	ENSGALG00000032626, ENSGALG00000032428, ENSGALG00000004480
11	8540001	8555000	15.0	86.779	ENSGALG00000004489
11	10440001	10455000	15.0	26.272	ENSGALG00000032365
11	10540001	10555000	15.0	59.748	ENSGALG00000004899
11	11560001	11575000	15.0	33.546	ENSGALG00000005251
11	17870001	17885000	15.0	30.457	ENSGALG00000031896
11	18310001	18325000	15.0	33.892	ENSGALG00000005872
12	3230001	3245000	15.0	41.809	ENSGALG00000029000, ENSGALG00000004661
12	5160001	5175000	15.0	44.772	ENSGALG00000005024
12	5880001	5890000	10.0	28.258	ENSGALG00000037711
12	10485001	10500000	15.0	25.271	ENSGALG00000029632
12	10515001	10525000	10.0	25.567	ENSGALG00000034289
13	1030001	1045000	15.0	48.056	ENSGALG00000000884
13	1260001	1275000	15.0	24.956	ENSGALG00000033641
13	4140001	4150000	10.0	24.429	ENSGALG00000001942
13	9150001	9165000	15.0	48.858	ENSGALG00000002878, ENSGALG00000002868
13	11530001	11545000	15.0	37.735	ENSGALG00000003886
14	8890001	8920000	30.0	60.614	ENSGALG00000007149
14	13745001	13760000	15.0	25.372	ENSGALG00000009341
14	13825001	13840000	15.0	70.385	ENSGALG00000009318
14	13965001	13980000	15.0	32.35	ENSGALG00000009217
15	6345001	6360000	15.0	26.833	ENSGALG00000043208, ENSGALG00000041821, ENSGALG00000036605
15	10390001	10405000	15.0	34.131	ENSGALG00000007732
15	11400001	11415000	15.0	118.649	ENSGALG00000008201, ENSGALG00000008206
17	4550001	4575000	25.0	50.593	ENSGALG00000006997
17	4580001	4590000	10.0	61.806	ENSGALG00000039255

17	7450001	7465000	15.0	24.377	ENSGALG00000002955
18	3500001	3535000	35.0	71.08	ENSGALG00000001577, ENSGALG00000017910, ENSGALG00000001603, ENSGALG00000034018
18	5930001	5945000	15.0	72.744	ENSGALG00000003033, ENSGALG00000038873
18	9030001	9045000	15.0	38.802	ENSGALG00000004413
19	905001	920000	15.0	46.191	ENSGALG00000043810
19	3635001	3650000	15.0	88.637	ENSGALG00000043223
20	2295001	2310000	15.0	28.67	ENSGALG00000003096
20	2405001	2420000	15.0	27.607	ENSGALG00000003106
20	3155001	3170000	15.0	51.235	ENSGALG00000037889
20	6585001	6595000	10.0	28.544	ENSGALG00000004663
20	6590001	6600000	10.0	28.544	ENSGALG00000004707
20	7015001	7030000	15.0	29.791	ENSGALG00000035809, ENSGALG00000045232
20	7065001	7075000	10.0	42.108	ENSGALG00000026542
21	3030001	3045000	15.0	24.153	ENSGALG00000002232
21	3420001	3435000	15.0	24.506	ENSGALG00000002583, ENSGALG00000002527
21	3565001	3575000	10.0	45.794	ENSGALG00000002616, ENSGALG00000043318, ENSGALG00000002637, ENSGALG00000002649
21	3670001	3685000	15.0	31.192	ENSGALG00000002726
21	4350001	4365000	15.0	40.837	ENSGALG00000003731
21	5280001	5295000	15.0	27.217	ENSGALG00000044962
22	2785001	2800000	15.0	24.667	ENSGALG00000014540
23	1815001	1830000	15.0	48.359	ENSGALG00000034910, ENSGALG00000028115, ENSGALG00000027909
23	1855001	1865000	10.0	40.062	ENSGALG00000000902, ENSGALG00000033047
24	3570001	3585000	15.0	35.069	ENSGALG00000018819
24	6100001	6125000	25.0	60.404	ENSGALG00000007848, ENSGALG00000007868
24	6135001	6145000	10.0	29.902	ENSGALG00000007874
25	1045001	1080000	35.0	41.751	ENSGALG00000024138, ENSGALG00000024136, ENSGALG00000032276, ENSGALG00000018882, ENSGALG00000029144, ENSGALG00000042032, ENSGALG00000031300,

					ENSGALG00000030486
26	815001	830000	15.0	30.489	ENSGALG00000000299
26	1345001	1355000	10.0	83.79	ENSGALG00000000394
26	1350001	1370000	20.0	83.79	ENSGALG00000000400
26	1505001	1520000	15.0	81.348	ENSGALG00000000477, ENSGALG00000000483
26	1575001	1590000	15.0	26.652	ENSGALG00000000533, ENSGALG00000028318
26	1735001	1750000	15.0	27.564	ENSGALG00000038399
27	655001	670000	15.0	37.312	ENSGALG00000032578, ENSGALG00000042952, ENSGALG00000038788, ENSGALG00000043374
27	745001	760000	15.0	37.078	ENSGALG00000038788, ENSGALG00000038568, ENSGALG00000039551, ENSGALG00000036968, ENSGALG00000030738, ENSGALG00000042950
27	945001	960000	15.0	30.783	ENSGALG00000038352, ENSGALG00000044718
27	1260001	1270000	10.0	39.056	ENSGALG00000001097
27	3185001	3200000	15.0	23.459	ENSGALG00000032702

Supplementary Table S10 Selected candidate genes by using XP-CLR

Gene ID
ENSGALG00000001577
ENSGALG00000016758
ENSGALG00000006760
ENSGALG00000006786
ENSGALG00000010658
ENSGALG00000002583
ENSGALG00000008201
ENSGALG00000004163
ENSGALG00000031751
ENSGALG00000013006
ENSGALG00000006446
ENSGALG00000027909
ENSGALG00000034910
ENSGALG00000010654
ENSGALG00000002878
ENSGALG00000015274

ENSGALG00000002649
ENSGALG00000043318
ENSGALG00000007868
ENSGALG00000030545
ENSGALG00000038385
ENSGALG00000002616
ENSGALG00000008265
ENSGALG00000014421
ENSGALG00000043208
ENSGALG00000031453
ENSGALG00000016759
ENSGALG00000008947
ENSGALG00000016828
ENSGALG00000006631
ENSGALG00000016861
ENSGALG00000045609
ENSGALG00000014210
ENSGALG00000037697
ENSGALG00000015378
ENSGALG00000030557
ENSGALG00000015303
ENSGALG00000013001
ENSGALG00000006431
ENSGALG00000015935
ENSGALG00000034392
ENSGALG00000038788
ENSGALG00000032578
ENSGALG00000042952
ENSGALG00000024138
ENSGALG00000007777
ENSGALG00000016548
ENSGALG00000006680
ENSGALG00000002467
ENSGALG00000030112
ENSGALG00000015297
ENSGALG00000003033
ENSGALG00000008759
ENSGALG00000008785
ENSGALG00000006997
ENSGALG00000045232
ENSGALG00000031713
ENSGALG00000000902
ENSGALG00000013187
ENSGALG00000015809
ENSGALG00000014525

ENSGALG00000043468
ENSGALG00000000299
ENSGALG00000000394
ENSGALG00000000400
ENSGALG00000000477
ENSGALG00000000483
ENSGALG00000000533
ENSGALG00000000884
ENSGALG00000001097
ENSGALG00000001942
ENSGALG00000002090
ENSGALG00000002232
ENSGALG00000002726
ENSGALG00000002744
ENSGALG00000002750
ENSGALG00000002955
ENSGALG00000003096
ENSGALG00000003106
ENSGALG00000003731
ENSGALG00000003886
ENSGALG00000003891
ENSGALG00000003899
ENSGALG00000004087
ENSGALG00000004171
ENSGALG00000004228
ENSGALG00000004288
ENSGALG00000004413
ENSGALG00000004480
ENSGALG00000004489
ENSGALG00000004661
ENSGALG00000004663
ENSGALG00000004707
ENSGALG00000004899
ENSGALG00000004966
ENSGALG00000005019
ENSGALG00000005024
ENSGALG00000005035
ENSGALG00000005251
ENSGALG00000005710
ENSGALG00000005872
ENSGALG00000006095
ENSGALG00000006112
ENSGALG00000006165
ENSGALG00000006302
ENSGALG00000006351

ENSGALG00000006583
ENSGALG00000006783
ENSGALG00000006939
ENSGALG00000006973
ENSGALG00000006983
ENSGALG00000007125
ENSGALG00000007140
ENSGALG00000007149
ENSGALG00000007417
ENSGALG00000007721
ENSGALG00000007732
ENSGALG00000007766
ENSGALG00000007817
ENSGALG00000007848
ENSGALG00000007874
ENSGALG00000007944
ENSGALG00000008006
ENSGALG00000008439
ENSGALG00000008474
ENSGALG00000008795
ENSGALG00000008890
ENSGALG00000008940
ENSGALG00000009217
ENSGALG00000009318
ENSGALG00000009325
ENSGALG00000009341
ENSGALG00000009458
ENSGALG00000009491
ENSGALG00000009601
ENSGALG00000009645
ENSGALG00000009730
ENSGALG00000009823
ENSGALG00000009838
ENSGALG00000009845
ENSGALG00000009850
ENSGALG00000009880
ENSGALG00000009995
ENSGALG00000010020
ENSGALG00000010057
ENSGALG00000010061
ENSGALG00000010078
ENSGALG00000010103
ENSGALG00000010131
ENSGALG00000010253
ENSGALG00000010311

ENSGALG00000010397
ENSGALG00000010567
ENSGALG00000010692
ENSGALG00000010718
ENSGALG00000010719
ENSGALG00000010764
ENSGALG00000010836
ENSGALG00000010840
ENSGALG00000010889
ENSGALG00000010935
ENSGALG00000011269
ENSGALG00000011271
ENSGALG00000011274
ENSGALG00000011297
ENSGALG00000011300
ENSGALG00000011562
ENSGALG00000011645
ENSGALG00000011854
ENSGALG00000011930
ENSGALG00000012024
ENSGALG00000012044
ENSGALG00000012115
ENSGALG00000012127
ENSGALG00000012129
ENSGALG00000012156
ENSGALG00000012190
ENSGALG00000012426
ENSGALG00000012559
ENSGALG00000012703
ENSGALG00000012739
ENSGALG00000012791
ENSGALG00000012792
ENSGALG00000012964
ENSGALG00000013061
ENSGALG00000013102
ENSGALG00000013124
ENSGALG00000013149
ENSGALG00000013154
ENSGALG00000013183
ENSGALG00000013193
ENSGALG00000013196
ENSGALG00000013200
ENSGALG00000013615
ENSGALG00000013674
ENSGALG00000013683

ENSGALG00000013708
ENSGALG00000013715
ENSGALG00000013874
ENSGALG00000013929
ENSGALG00000013932
ENSGALG00000014144
ENSGALG00000014178
ENSGALG00000014192
ENSGALG00000014540
ENSGALG00000015198
ENSGALG00000015258
ENSGALG00000015267
ENSGALG00000015389
ENSGALG00000015410
ENSGALG00000015733
ENSGALG00000015770
ENSGALG00000015786
ENSGALG00000015966
ENSGALG00000016044
ENSGALG00000016047
ENSGALG00000016236
ENSGALG00000016281
ENSGALG00000016287
ENSGALG00000016292
ENSGALG00000016316
ENSGALG00000016320
ENSGALG00000016382
ENSGALG00000016392
ENSGALG00000016445
ENSGALG00000016463
ENSGALG00000016649
ENSGALG00000016686
ENSGALG00000016705
ENSGALG00000016817
ENSGALG00000016868
ENSGALG00000016902
ENSGALG00000016984
ENSGALG00000017068
ENSGALG00000017125
ENSGALG00000017283
ENSGALG00000018819
ENSGALG00000019157
ENSGALG00000019221
ENSGALG00000019300
ENSGALG00000019527

ENSGALG00000020775
ENSGALG00000022875
ENSGALG00000024136
ENSGALG00000025484
ENSGALG00000025546
ENSGALG00000025619
ENSGALG00000025630
ENSGALG00000026542
ENSGALG00000026661
ENSGALG00000027620
ENSGALG00000028250
ENSGALG00000028318
ENSGALG00000028744
ENSGALG00000029000
ENSGALG00000029190
ENSGALG00000029379
ENSGALG00000029632
ENSGALG00000029794
ENSGALG00000030030
ENSGALG00000030357
ENSGALG00000030588
ENSGALG00000030680
ENSGALG00000030872
ENSGALG00000030944
ENSGALG00000030991
ENSGALG00000031387
ENSGALG00000031503
ENSGALG00000031547
ENSGALG00000031758
ENSGALG00000031896
ENSGALG00000032053
ENSGALG00000032103
ENSGALG00000032365
ENSGALG00000032554
ENSGALG00000032577
ENSGALG00000032626
ENSGALG00000032640
ENSGALG00000032651
ENSGALG00000032702
ENSGALG00000032850
ENSGALG00000032868
ENSGALG00000033089
ENSGALG00000033099
ENSGALG00000033170
ENSGALG00000033533

ENSGALG00000033641
ENSGALG00000033880
ENSGALG00000034003
ENSGALG00000034081
ENSGALG00000034188
ENSGALG00000034289
ENSGALG00000034616
ENSGALG00000035337
ENSGALG00000035769
ENSGALG00000035809
ENSGALG00000036110
ENSGALG00000036567
ENSGALG00000036597
ENSGALG00000036693
ENSGALG00000037014
ENSGALG00000037109
ENSGALG00000037336
ENSGALG00000037423
ENSGALG00000037581
ENSGALG00000037626
ENSGALG00000037681
ENSGALG00000037711
ENSGALG00000037889
ENSGALG00000038062
ENSGALG00000038160
ENSGALG00000038352
ENSGALG00000038399
ENSGALG00000038439
ENSGALG00000038688
ENSGALG00000038733
ENSGALG00000038782
ENSGALG00000038924
ENSGALG00000039165
ENSGALG00000039224
ENSGALG00000039238
ENSGALG00000039255
ENSGALG00000039327
ENSGALG00000039349
ENSGALG00000039935
ENSGALG00000040491
ENSGALG00000040607
ENSGALG00000040891
ENSGALG00000040945
ENSGALG00000040948
ENSGALG00000041094

ENSGALG00000041312
ENSGALG00000041356
ENSGALG00000041373
ENSGALG00000041571
ENSGALG00000041575
ENSGALG00000041701
ENSGALG00000042076
ENSGALG00000042259
ENSGALG00000042484
ENSGALG00000042795
ENSGALG00000042836
ENSGALG00000043172
ENSGALG00000043173
ENSGALG00000043223
ENSGALG00000043428
ENSGALG00000043439
ENSGALG00000043524
ENSGALG00000043800
ENSGALG00000043810
ENSGALG00000044225
ENSGALG00000044962
ENSGALG00000045327
ENSGALG00000045557
ENSGALG00000045596
ENSGALG00000045907
ENSGALG00000001603
ENSGALG00000002527
ENSGALG00000002637
ENSGALG00000002868
ENSGALG00000004173
ENSGALG00000006611
ENSGALG00000006773
ENSGALG00000007841
ENSGALG00000008206
ENSGALG00000008918
ENSGALG00000013005
ENSGALG00000014425
ENSGALG00000014526
ENSGALG00000015805
ENSGALG00000015937
ENSGALG00000016549
ENSGALG00000016757
ENSGALG00000016862
ENSGALG00000017910
ENSGALG00000018330

ENSGALG00000024572
ENSGALG00000025103
ENSGALG00000025806
ENSGALG00000025941
ENSGALG00000027236
ENSGALG00000028115
ENSGALG00000028297
ENSGALG00000030486
ENSGALG00000030738
ENSGALG00000031300
ENSGALG00000032712
ENSGALG00000033047
ENSGALG00000033634
ENSGALG00000034018
ENSGALG00000034312
ENSGALG00000035699
ENSGALG00000035909
ENSGALG00000036465
ENSGALG00000036605
ENSGALG00000036968
ENSGALG00000038395
ENSGALG00000038873
ENSGALG00000039551
ENSGALG00000039898
ENSGALG00000041631
ENSGALG00000041821
ENSGALG00000042950
ENSGALG00000043079
ENSGALG00000043420
ENSGALG00000043440
ENSGALG00000045042

Supplementary Table S11 Select candidate genes in the union of log2 θπ ratio, F_{ST} and XP-CLR

**Gene ID**
ENSGALG00000000311
ENSGALG00000000394
ENSGALG00000000400
ENSGALG00000000401
ENSGALG00000000477
ENSGALG00000000483
ENSGALG00000000497
ENSGALG00000000504

ENSGALG00000000511
ENSGALG00000001046
ENSGALG00000001577
ENSGALG00000001603
ENSGALG00000001753
ENSGALG00000001942
ENSGALG00000002080
ENSGALG00000002119
ENSGALG00000002134
ENSGALG00000002200
ENSGALG00000002616
ENSGALG00000002637
ENSGALG00000002649
ENSGALG00000002671
ENSGALG00000002726
ENSGALG00000002797
ENSGALG00000002967
ENSGALG00000002980
ENSGALG00000003135
ENSGALG00000003163
ENSGALG00000003201
ENSGALG00000003224
ENSGALG00000003258
ENSGALG00000003273
ENSGALG00000003293
ENSGALG00000003302
ENSGALG00000003309
ENSGALG00000003891
ENSGALG00000003969
ENSGALG00000003991
ENSGALG00000004005
ENSGALG00000004034
ENSGALG00000004087
ENSGALG00000004196
ENSGALG00000004228
ENSGALG00000004474
ENSGALG00000004480
ENSGALG00000004489
ENSGALG00000004621
ENSGALG00000004625
ENSGALG00000004650
ENSGALG00000004663
ENSGALG00000004707
ENSGALG00000004736
ENSGALG00000004899

ENSGALG00000004966
ENSGALG00000005000
ENSGALG00000005002
ENSGALG00000005019
ENSGALG00000005035
ENSGALG00000005065
ENSGALG00000005257
ENSGALG00000005277
ENSGALG00000005280
ENSGALG00000005284
ENSGALG00000005347
ENSGALG00000005470
ENSGALG00000005478
ENSGALG00000005509
ENSGALG00000005552
ENSGALG00000005857
ENSGALG00000005867
ENSGALG00000006034
ENSGALG00000006048
ENSGALG00000006064
ENSGALG00000006095
ENSGALG00000006165
ENSGALG00000006168
ENSGALG00000006174
ENSGALG00000006302
ENSGALG00000006331
ENSGALG00000006343
ENSGALG00000006351
ENSGALG00000006427
ENSGALG00000006431
ENSGALG00000006446
ENSGALG00000006502
ENSGALG00000006783
ENSGALG00000006983
ENSGALG00000006997
ENSGALG00000007125
ENSGALG00000007130
ENSGALG00000007133
ENSGALG00000007140
ENSGALG00000007149
ENSGALG00000007211
ENSGALG00000007431
ENSGALG00000007777
ENSGALG00000007841
ENSGALG00000007848

ENSGALG00000007868
ENSGALG00000007874
ENSGALG00000007878
ENSGALG00000007882
ENSGALG00000007885
ENSGALG00000007904
ENSGALG00000007929
ENSGALG00000007963
ENSGALG00000008470
ENSGALG00000008474
ENSGALG00000008486
ENSGALG00000008497
ENSGALG00000008988
ENSGALG00000009012
ENSGALG00000009153
ENSGALG00000009325
ENSGALG00000009487
ENSGALG00000009491
ENSGALG00000009528
ENSGALG00000009554
ENSGALG00000009560
ENSGALG00000009645
ENSGALG00000009650
ENSGALG00000009698
ENSGALG00000009838
ENSGALG00000009844
ENSGALG00000009845
ENSGALG00000009847
ENSGALG00000009850
ENSGALG00000009880
ENSGALG00000010057
ENSGALG00000010068
ENSGALG00000010103
ENSGALG00000010131
ENSGALG00000010654
ENSGALG00000010658
ENSGALG00000010659
ENSGALG00000010661
ENSGALG00000010692
ENSGALG00000010718
ENSGALG00000010764
ENSGALG00000010836
ENSGALG00000010840
ENSGALG00000010889
ENSGALG00000010935

ENSGALG00000010943
ENSGALG00000011124
ENSGALG00000011269
ENSGALG00000011270
ENSGALG00000011271
ENSGALG00000011285
ENSGALG00000011295
ENSGALG00000011297
ENSGALG00000011394
ENSGALG00000011499
ENSGALG00000011854
ENSGALG00000011930
ENSGALG00000012044
ENSGALG00000012127
ENSGALG00000012156
ENSGALG00000012190
ENSGALG00000012264
ENSGALG00000012426
ENSGALG00000012715
ENSGALG00000012739
ENSGALG00000012755
ENSGALG00000012760
ENSGALG00000012761
ENSGALG00000012791
ENSGALG00000012941
ENSGALG00000012964
ENSGALG00000013102
ENSGALG00000013180
ENSGALG00000013183
ENSGALG00000013187
ENSGALG00000013189
ENSGALG00000013196
ENSGALG00000013197
ENSGALG00000013200
ENSGALG00000013615
ENSGALG00000013617
ENSGALG00000013715
ENSGALG00000013929
ENSGALG00000013964
ENSGALG00000013968
ENSGALG00000014078
ENSGALG00000014144
ENSGALG00000014178
ENSGALG00000014192
ENSGALG00000014276

ENSGALG00000014329
ENSGALG00000015198
ENSGALG00000015254
ENSGALG00000015267
ENSGALG00000015274
ENSGALG00000015410
ENSGALG00000015770
ENSGALG00000015773
ENSGALG00000015783
ENSGALG00000015786
ENSGALG00000015904
ENSGALG00000015906
ENSGALG00000015908
ENSGALG00000015935
ENSGALG00000015937
ENSGALG00000015939
ENSGALG00000016044
ENSGALG00000016054
ENSGALG00000016276
ENSGALG00000016382
ENSGALG00000016388
ENSGALG00000016389
ENSGALG00000016391
ENSGALG00000016392
ENSGALG00000016828
ENSGALG00000016860
ENSGALG00000016861
ENSGALG00000016862
ENSGALG00000016863
ENSGALG00000016868
ENSGALG00000016902
ENSGALG00000017111
ENSGALG00000017112
ENSGALG00000017228
ENSGALG00000017271
ENSGALG00000017272
ENSGALG00000017280
ENSGALG00000017282
ENSGALG00000017283
ENSGALG00000017860
ENSGALG00000017910
ENSGALG00000017930
ENSGALG00000018330
ENSGALG00000018882
ENSGALG00000019157

ENSGALG00000019361
ENSGALG00000019527
ENSGALG00000020049
ENSGALG00000020160
ENSGALG00000020201
ENSGALG00000020939
ENSGALG00000023584
ENSGALG00000023761
ENSGALG00000023824
ENSGALG00000024136
ENSGALG00000024138
ENSGALG00000024481
ENSGALG00000024572
ENSGALG00000025103
ENSGALG00000025557
ENSGALG00000025619
ENSGALG00000025796
ENSGALG00000025941
ENSGALG00000026264
ENSGALG00000026372
ENSGALG00000026409
ENSGALG00000026661
ENSGALG00000026761
ENSGALG00000026808
ENSGALG00000026901
ENSGALG00000027620
ENSGALG00000027788
ENSGALG00000027816
ENSGALG00000027872
ENSGALG00000027893
ENSGALG00000028250
ENSGALG00000028297
ENSGALG00000028986
ENSGALG00000029132
ENSGALG00000029144
ENSGALG00000029190
ENSGALG00000029322
ENSGALG00000029372
ENSGALG00000029580
ENSGALG00000029785
ENSGALG00000029829
ENSGALG00000029853
ENSGALG00000029961
ENSGALG00000029984
ENSGALG00000030006

ENSGALG00000030061
ENSGALG00000030112
ENSGALG00000030235
ENSGALG00000030486
ENSGALG00000030532
ENSGALG00000030557
ENSGALG00000030563
ENSGALG00000030614
ENSGALG00000030680
ENSGALG00000030791
ENSGALG00000030836
ENSGALG00000030867
ENSGALG00000030991
ENSGALG00000031157
ENSGALG00000031268
ENSGALG00000031294
ENSGALG00000031300
ENSGALG00000031323
ENSGALG00000031503
ENSGALG00000031713
ENSGALG00000031751
ENSGALG00000031758
ENSGALG00000031808
ENSGALG00000031929
ENSGALG00000032164
ENSGALG00000032211
ENSGALG00000032234
ENSGALG00000032276
ENSGALG00000032339
ENSGALG00000032428
ENSGALG00000032626
ENSGALG00000032640
ENSGALG00000032651
ENSGALG00000032683
ENSGALG00000032687
ENSGALG00000033015
ENSGALG00000033099
ENSGALG00000033204
ENSGALG00000033407
ENSGALG00000033482
ENSGALG00000033533
ENSGALG00000033635
ENSGALG00000033641
ENSGALG00000033880
ENSGALG00000033883

ENSGALG00000033942
ENSGALG00000034003
ENSGALG00000034018
ENSGALG00000034188
ENSGALG00000034232
ENSGALG00000034312
ENSGALG00000034616
ENSGALG00000034628
ENSGALG00000034962
ENSGALG00000034994
ENSGALG00000035126
ENSGALG00000035175
ENSGALG00000035602
ENSGALG00000035665
ENSGALG00000035769
ENSGALG00000035886
ENSGALG00000035909
ENSGALG00000035959
ENSGALG00000036110
ENSGALG00000036112
ENSGALG00000036133
ENSGALG00000036149
ENSGALG00000036190
ENSGALG00000036222
ENSGALG00000036262
ENSGALG00000036567
ENSGALG00000036693
ENSGALG00000036730
ENSGALG00000036742
ENSGALG00000036942
ENSGALG00000036951
ENSGALG00000036972
ENSGALG00000037267
ENSGALG00000037309
ENSGALG00000037336
ENSGALG00000037581
ENSGALG00000037587
ENSGALG00000037613
ENSGALG00000037656
ENSGALG00000037697
ENSGALG00000037889
ENSGALG00000037995
ENSGALG00000038160
ENSGALG00000038385
ENSGALG00000038395

ENSGALG00000038439
ENSGALG00000038607
ENSGALG00000038924
ENSGALG00000038929
ENSGALG00000039004
ENSGALG00000039034
ENSGALG00000039168
ENSGALG00000039238
ENSGALG00000039255
ENSGALG00000039442
ENSGALG00000039534
ENSGALG00000039935
ENSGALG00000039997
ENSGALG00000040266
ENSGALG00000040421
ENSGALG00000040444
ENSGALG00000040607
ENSGALG00000040645
ENSGALG00000040681
ENSGALG00000040891
ENSGALG00000040898
ENSGALG00000040948
ENSGALG00000041086
ENSGALG00000041094
ENSGALG00000041312
ENSGALG00000041346
ENSGALG00000041356
ENSGALG00000041373
ENSGALG00000041571
ENSGALG00000041873
ENSGALG00000041941
ENSGALG00000041972
ENSGALG00000042032
ENSGALG00000042231
ENSGALG00000042427
ENSGALG00000042507
ENSGALG00000042678
ENSGALG00000042836
ENSGALG00000042853
ENSGALG00000042905
ENSGALG00000043079
ENSGALG00000043172
ENSGALG00000043173
ENSGALG00000043204
ENSGALG00000043207

ENSGALG00000043258
ENSGALG00000043265
ENSGALG00000043318
ENSGALG00000043440
ENSGALG00000043800
ENSGALG00000043837
ENSGALG00000043856
ENSGALG00000044610
ENSGALG00000044780
ENSGALG00000044890
ENSGALG00000044892
ENSGALG00000044908
ENSGALG00000045026
ENSGALG00000045129
ENSGALG00000045240
ENSGALG00000045466
ENSGALG00000045609
ENSGALG00000045907
ENSGALG00000045908
ENSGALG00000046009
ENSGALG00000046044
ENSGALG00000046372
ENSGALG00000046392
ENSGALG00000016758
ENSGALG00000006760
ENSGALG00000006786
ENSGALG00000002583
ENSGALG00000008201
ENSGALG00000004163
ENSGALG00000013006
ENSGALG00000027909
ENSGALG00000034910
ENSGALG00000002878
ENSGALG00000030545
ENSGALG00000008265
ENSGALG00000014421
ENSGALG00000043208
ENSGALG00000031453
ENSGALG00000016759
ENSGALG00000008947
ENSGALG00000006631
ENSGALG00000014210
ENSGALG00000015378
ENSGALG00000015303
ENSGALG00000013001

ENSGALG00000034392
ENSGALG00000038788
ENSGALG00000032578
ENSGALG00000042952
ENSGALG00000016548
ENSGALG00000006680
ENSGALG00000002467
ENSGALG00000015297
ENSGALG00000003033
ENSGALG00000008759
ENSGALG00000008785
ENSGALG00000045232
ENSGALG00000000902
ENSGALG00000015809
ENSGALG00000014525
ENSGALG00000043468
ENSGALG00000000299
ENSGALG00000000533
ENSGALG00000000884
ENSGALG00000001097
ENSGALG00000002090
ENSGALG00000002232
ENSGALG00000002744
ENSGALG00000002750
ENSGALG00000002955
ENSGALG00000003096
ENSGALG00000003106
ENSGALG00000003731
ENSGALG00000003886
ENSGALG00000003899
ENSGALG00000004171
ENSGALG00000004288
ENSGALG00000004413
ENSGALG00000004661
ENSGALG00000005024
ENSGALG00000005251
ENSGALG00000005710
ENSGALG00000005872
ENSGALG00000006112
ENSGALG00000006583
ENSGALG00000006939
ENSGALG00000006973
ENSGALG00000007417
ENSGALG00000007721
ENSGALG00000007732

ENSGALG00000007766
ENSGALG00000007817
ENSGALG00000007944
ENSGALG00000008006
ENSGALG00000008439
ENSGALG00000008795
ENSGALG00000008890
ENSGALG00000008940
ENSGALG00000009217
ENSGALG00000009318
ENSGALG00000009341
ENSGALG00000009458
ENSGALG00000009601
ENSGALG00000009730
ENSGALG00000009823
ENSGALG00000009995
ENSGALG00000010020
ENSGALG00000010061
ENSGALG00000010078
ENSGALG00000010253
ENSGALG00000010311
ENSGALG00000010397
ENSGALG00000010567
ENSGALG00000010719
ENSGALG00000011274
ENSGALG00000011300
ENSGALG00000011562
ENSGALG00000011645
ENSGALG00000012024
ENSGALG00000012115
ENSGALG00000012129
ENSGALG00000012559
ENSGALG00000012703
ENSGALG00000012792
ENSGALG00000013061
ENSGALG00000013124
ENSGALG00000013149
ENSGALG00000013154
ENSGALG00000013193
ENSGALG00000013674
ENSGALG00000013683
ENSGALG00000013708
ENSGALG00000013874
ENSGALG00000013932
ENSGALG00000014540

ENSGALG00000015258
ENSGALG00000015389
ENSGALG00000015733
ENSGALG00000015966
ENSGALG00000016047
ENSGALG00000016236
ENSGALG00000016281
ENSGALG00000016287
ENSGALG00000016292
ENSGALG00000016316
ENSGALG00000016320
ENSGALG00000016445
ENSGALG00000016463
ENSGALG00000016649
ENSGALG00000016686
ENSGALG00000016705
ENSGALG00000016817
ENSGALG00000016984
ENSGALG00000017068
ENSGALG00000017125
ENSGALG00000018819
ENSGALG00000019221
ENSGALG00000019300
ENSGALG00000020775
ENSGALG00000022875
ENSGALG00000025484
ENSGALG00000025546
ENSGALG00000025630
ENSGALG00000026542
ENSGALG00000028318
ENSGALG00000028744
ENSGALG00000029000
ENSGALG00000029379
ENSGALG00000029632
ENSGALG00000029794
ENSGALG00000030030
ENSGALG00000030357
ENSGALG00000030588
ENSGALG00000030872
ENSGALG00000030944
ENSGALG00000031387
ENSGALG00000031547
ENSGALG00000031896
ENSGALG00000032053
ENSGALG00000032103

ENSGALG00000032365
ENSGALG00000032554
ENSGALG00000032577
ENSGALG00000032702
ENSGALG00000032850
ENSGALG00000032868
ENSGALG00000033089
ENSGALG00000033170
ENSGALG00000034081
ENSGALG00000034289
ENSGALG00000035337
ENSGALG00000035809
ENSGALG00000036597
ENSGALG00000037014
ENSGALG00000037109
ENSGALG00000037423
ENSGALG00000037626
ENSGALG00000037681
ENSGALG00000037711
ENSGALG00000038062
ENSGALG00000038352
ENSGALG00000038399
ENSGALG00000038688
ENSGALG00000038733
ENSGALG00000038782
ENSGALG00000039165
ENSGALG00000039224
ENSGALG00000039327
ENSGALG00000039349
ENSGALG00000040491
ENSGALG00000040945
ENSGALG00000041575
ENSGALG00000041701
ENSGALG00000042076
ENSGALG00000042259
ENSGALG00000042484
ENSGALG00000042795
ENSGALG00000043223
ENSGALG00000043428
ENSGALG00000043439
ENSGALG00000043524
ENSGALG00000043810
ENSGALG00000044225
ENSGALG00000044962
ENSGALG00000045327

ENSGALG00000045557
ENSGALG00000045596
ENSGALG00000002527
ENSGALG00000002868
ENSGALG00000004173
ENSGALG00000006611
ENSGALG00000006773
ENSGALG00000008206
ENSGALG00000008918
ENSGALG00000013005
ENSGALG00000014425
ENSGALG00000014526
ENSGALG00000015805
ENSGALG00000016549
ENSGALG00000016757
ENSGALG00000025806
ENSGALG00000027236
ENSGALG00000028115
ENSGALG00000030738
ENSGALG00000032712
ENSGALG00000033047
ENSGALG00000033634
ENSGALG00000035699
ENSGALG00000036465
ENSGALG00000036605
ENSGALG00000036968
ENSGALG00000038873
ENSGALG00000039551
ENSGALG00000039898
ENSGALG00000041631
ENSGALG00000041821
ENSGALG00000042950
ENSGALG00000043420
ENSGALG00000045042

Supplementary Table S12 Select candidate genes overlapped with the results from log2 θπ ratio, F_{ST} and XP-CLR

Gene ID
ENSGALG00000000394
ENSGALG00000000400
ENSGALG00000000477
ENSGALG00000000483
ENSGALG00000001577
ENSGALG00000001603
ENSGALG00000001942
ENSGALG00000002616
ENSGALG00000002637
ENSGALG00000002649
ENSGALG00000002726
ENSGALG00000003891
ENSGALG00000004087
ENSGALG00000004228
ENSGALG00000004480
ENSGALG00000004489
ENSGALG00000004663
ENSGALG00000004707
ENSGALG00000004899
ENSGALG00000004966
ENSGALG00000005019
ENSGALG00000005035
ENSGALG00000006095
ENSGALG00000006165
ENSGALG00000006302
ENSGALG00000006351
ENSGALG00000006431
ENSGALG00000006446
ENSGALG00000006783
ENSGALG00000006983
ENSGALG00000006997
ENSGALG00000007125
ENSGALG00000007140
ENSGALG00000007149
ENSGALG00000007777
ENSGALG00000007841
ENSGALG00000007848
ENSGALG00000007868
ENSGALG00000007874
ENSGALG00000008474
ENSGALG00000009325

ENSGALG00000009491
ENSGALG00000009645
ENSGALG00000009838
ENSGALG00000009845
ENSGALG00000009850
ENSGALG00000009880
ENSGALG00000010057
ENSGALG00000010103
ENSGALG00000010131
ENSGALG00000010654
ENSGALG00000010658
ENSGALG00000010692
ENSGALG00000010718
ENSGALG00000010764
ENSGALG00000010836
ENSGALG00000010840
ENSGALG00000010889
ENSGALG00000010935
ENSGALG00000011269
ENSGALG00000011271
ENSGALG00000011297
ENSGALG00000011854
ENSGALG00000011930
ENSGALG00000012044
ENSGALG00000012127
ENSGALG00000012156
ENSGALG00000012190
ENSGALG00000012426
ENSGALG00000012739
ENSGALG00000012791
ENSGALG00000012964
ENSGALG00000013102
ENSGALG00000013183
ENSGALG00000013187
ENSGALG00000013196
ENSGALG00000013200
ENSGALG00000013615
ENSGALG00000013715
ENSGALG00000013929
ENSGALG00000014144
ENSGALG00000014178
ENSGALG00000014192
ENSGALG00000015198
ENSGALG00000015267
ENSGALG00000015274

ENSGALG00000015410
ENSGALG00000015770
ENSGALG00000015786
ENSGALG00000015935
ENSGALG00000015937
ENSGALG00000016044
ENSGALG00000016382
ENSGALG00000016392
ENSGALG00000016828
ENSGALG00000016861
ENSGALG00000016862
ENSGALG00000016868
ENSGALG00000016902
ENSGALG00000017283
ENSGALG00000017910
ENSGALG00000018330
ENSGALG00000019157
ENSGALG00000019527
ENSGALG00000024136
ENSGALG00000024138
ENSGALG00000024572
ENSGALG00000025103
ENSGALG00000025619
ENSGALG00000025941
ENSGALG00000026661
ENSGALG00000027620
ENSGALG00000028250
ENSGALG00000028297
ENSGALG00000029190
ENSGALG00000030112
ENSGALG00000030486
ENSGALG00000030557
ENSGALG00000030680
ENSGALG00000030991
ENSGALG00000031300
ENSGALG00000031503
ENSGALG00000031713
ENSGALG00000031751
ENSGALG00000031758
ENSGALG00000032626
ENSGALG00000032640
ENSGALG00000032651
ENSGALG00000033099
ENSGALG00000033533
ENSGALG00000033641

ENSGALG00000033880
ENSGALG00000034003
ENSGALG00000034018
ENSGALG00000034188
ENSGALG00000034312
ENSGALG00000034616
ENSGALG00000035769
ENSGALG00000035909
ENSGALG00000036110
ENSGALG00000036567
ENSGALG00000036693
ENSGALG00000037336
ENSGALG00000037581
ENSGALG00000037697
ENSGALG00000037889
ENSGALG00000038160
ENSGALG00000038385
ENSGALG00000038395
ENSGALG00000038439
ENSGALG00000038924
ENSGALG00000039238
ENSGALG00000039255
ENSGALG00000039935
ENSGALG00000040607
ENSGALG00000040891
ENSGALG00000040948
ENSGALG00000041094
ENSGALG00000041312
ENSGALG00000041356
ENSGALG00000041373
ENSGALG00000041571
ENSGALG00000042836
ENSGALG00000043079
ENSGALG00000043172
ENSGALG00000043173
ENSGALG00000043318
ENSGALG00000043440
ENSGALG00000043800
ENSGALG00000045609
ENSGALG00000045907

Supplementary Table S13 The functional analysis of candidate genes (log2 θπ ratio and F_{ST} method) using DAVID

GO accession	Description	P-value	Gene (#)	Genes Symbol
GO-BP:0032012	Regulation of ARF protein signal transduction	1.98E-02	3	*ARFGEF2, FBXO8, CYTH3*
GO-CC:0043679	Axon terminus	2.57E-02	3	*NRP1, KCNC4, KCNA1*
GO-MF:0015171	Amino acid transmembrane transporter activity	2.74E-02	3	*SLC38A2, SLC6A17, SLC6A18*
GO-CC:0005578	Proteinaceous extracellular matrix	3.06E-02	7	*ADAMTS18, COL12A1, EPYC, GPC5, KERA, LUM, VWF*
KEGG-pathway:00590	Arachidonic acid metabolism	3.27E-02	4	*ALOX5, CYP2J23, PLA2G4A, TBXAS1*
GO-BP:0014904	Myotube cell development	3.37E-02	2	*IGF1, NFATC2*
GO-MF:0005328	Neurotransmitter:sodium symporter activity	3.47E-02	3	*SLC6A17, SLC6A18, SLC6A19*
GO-MF:0005086	ARF guanyl-nucleotide exchange factor activity	3.86E-02	3	*ARFGEF2, FBXO8, CYTH3*

Supplementary Table S14 The functional analysis of candidate genes (XP-CLR method) using DAVID

GO accession	Description	P-value	Gene (#)	Genes Symbol
KEGG-pathway:04115	P53 signaling pathway	7.62E-03	5	*FAS, CDKN1A, CCND2, SFN, SHISA5*
GO-MF:0016887	ATPase activity	1.78E-02	6	*KIF7, MDN1, KIF1B, KIF6, OLA1, DNAH7*
GO-BP:0032012	Regulation of ARF protein signal transduction	2.26E-02	3	*CYTH3, ARFGEF2, FBXO8*
GO-CC:0042383	Sarcolemma	2.39E-02	4	*BVES, DMD, SGCD, DTNBP1*
GO-BP:0042771	Intrinsic apoptotic signaling pathway in response to DNA damage by p53 class mediator	2.97E-02	3	*CDKN1A, SHISA5,HIPK2*
GO-MF:0003777	Microtubule motor activity	3.50E-02	4	*KIF7, KIF1B, KIF6, DNAH7*
GO-BP:0030325	Adrenal gland development	3.76E-02	3	*WT1, NR0B1, PDGFRA*
GO-MF:0005086	ARF guanyl-nucleotide exchange factor activity	4.29E-02	3	*CYTH3, ARFGEF2, FBXO8*

Supplementary Table S15 Information of the Read Archive (SRA) uploaded on the NCBI

**Accession**	**Library name**	**Sample information**
SRX2196364	DG	Dagu
SRX2196363	EM	Emei Black
SRX2196355	HT	Hetian
SRX2196356	LH	Luhua
SRX2196365	LS	Longsheng
SRX2196366	NX1	Nixi
SRX2196367	NX2	Nixi
SRX2196368	NX3	Nixi
SRX2196369	NY1	Niya
SRX2196370	NY2	Niya
SRX2196371	NY3	Niya
SRX6424288	NY4	Niya
SRX6424287	NY5	Niya
SRX6424290	NY6	Niya
SRX6424289	NY7	Niya
SRX6424284	NY8	Niya
SRX6424283	NY9	Niya
SRX6424286	NY10	Niya
SRX6424285	NY11	Niya
SRX6424282	NY12	Niya
SRX6424281	NY13	Niya
SRX6424292	NY14	Niya
SRX6424291	NY15	Niya
SRX2196372	P1	Piao
SRX2196357	P2	Piao
SRX2196358	P3	Piao
SRX2196359	QX	Qianxiang
SRX2196360	QY	Qingyuan
SRX2196361	TY	Taoyuan
SRX2196362	XJ	Xianju
